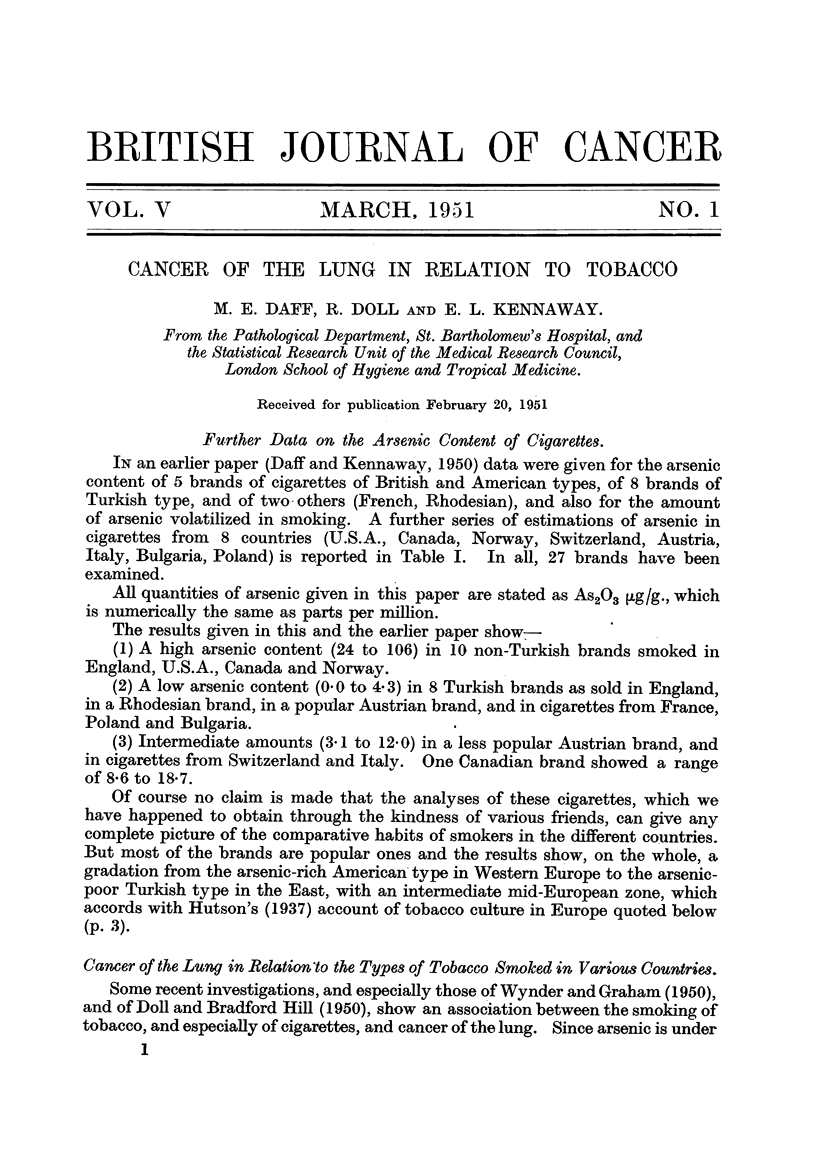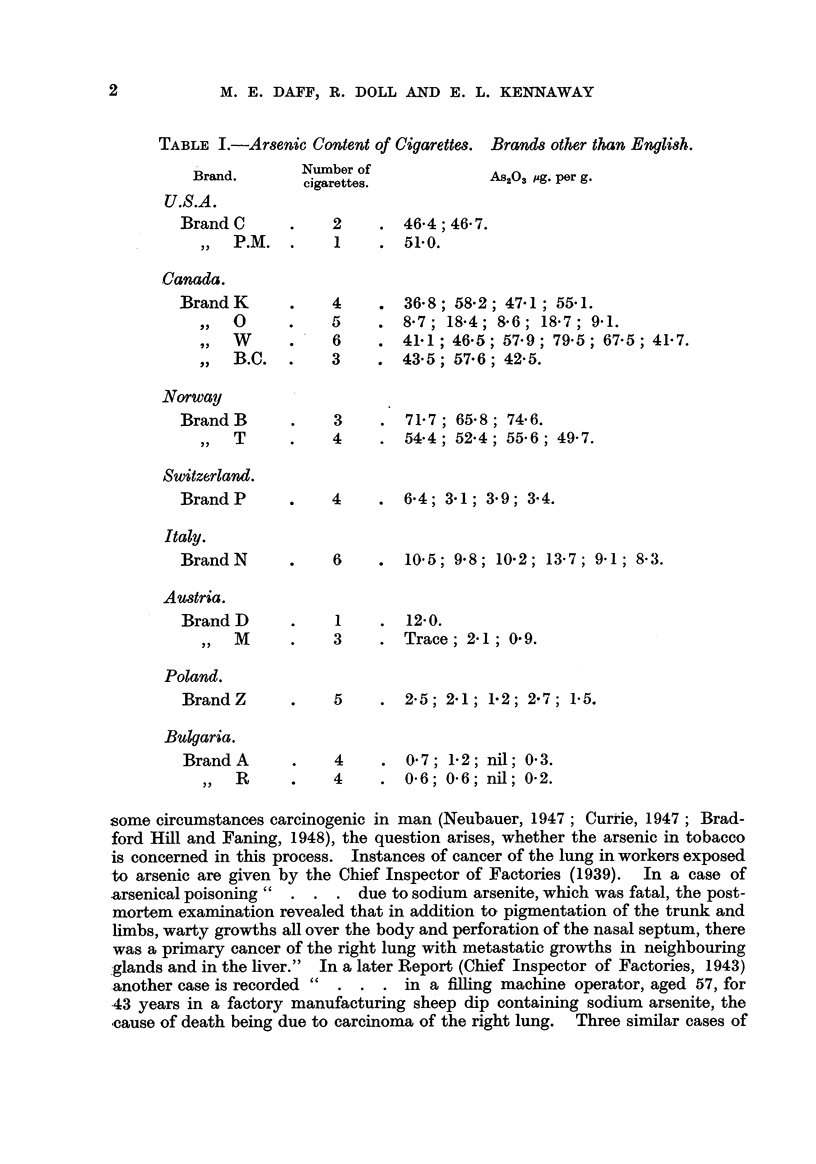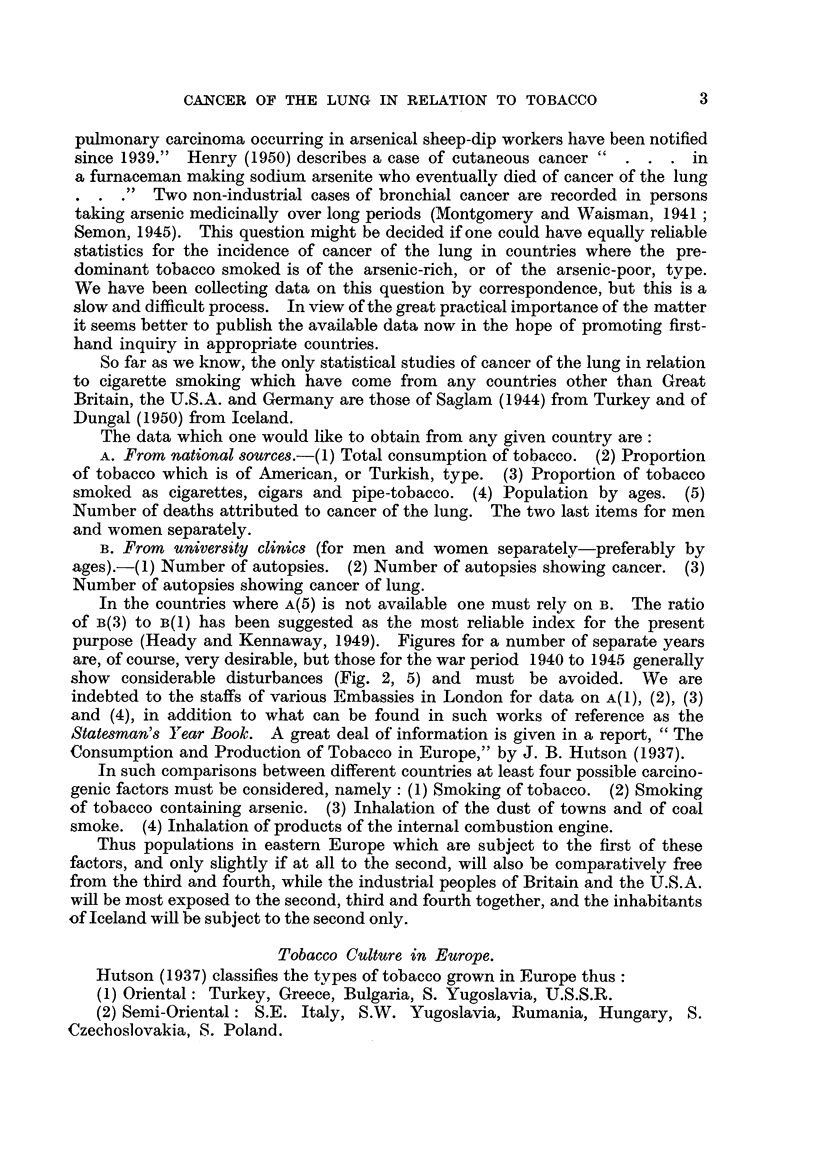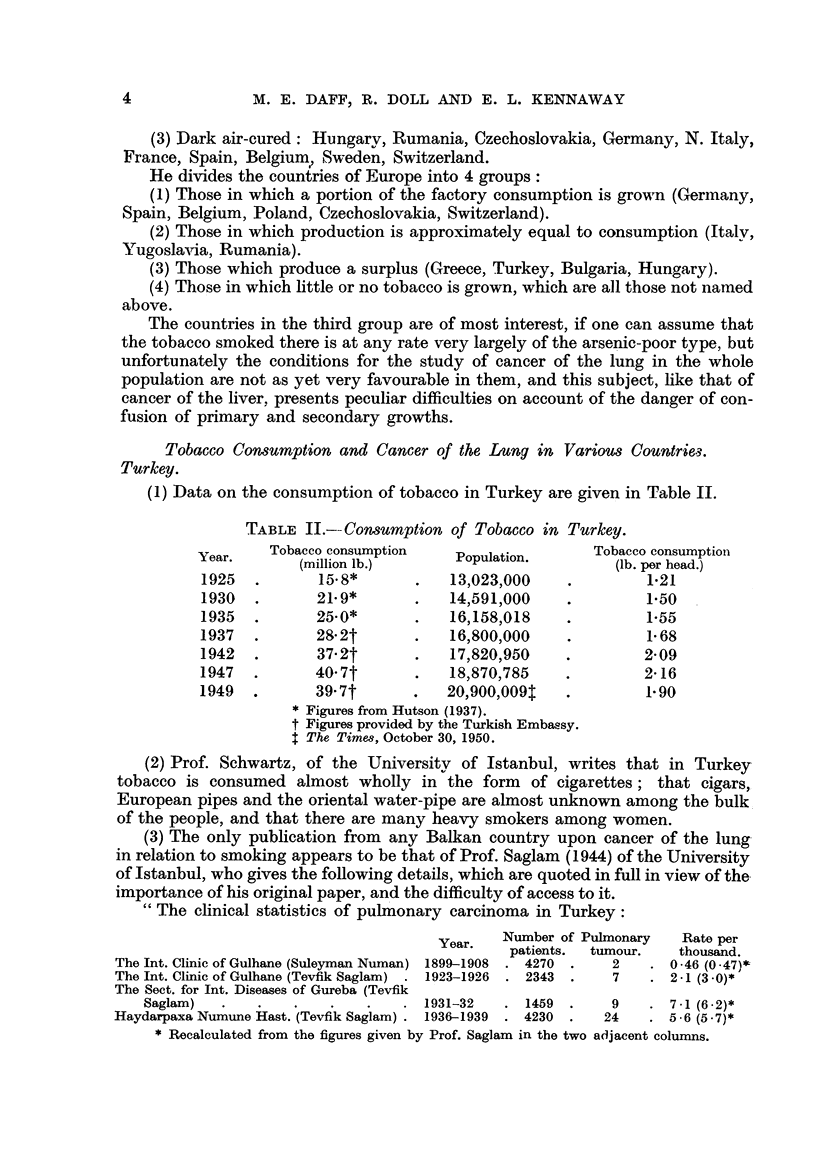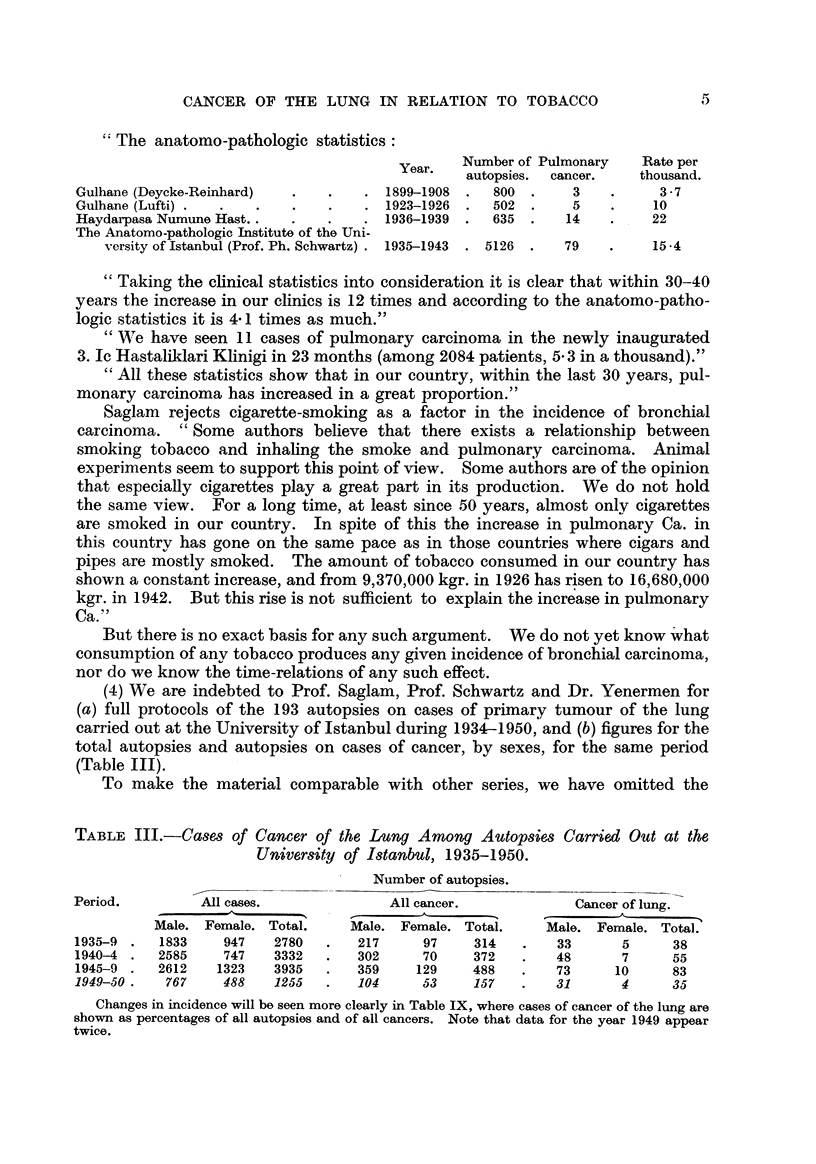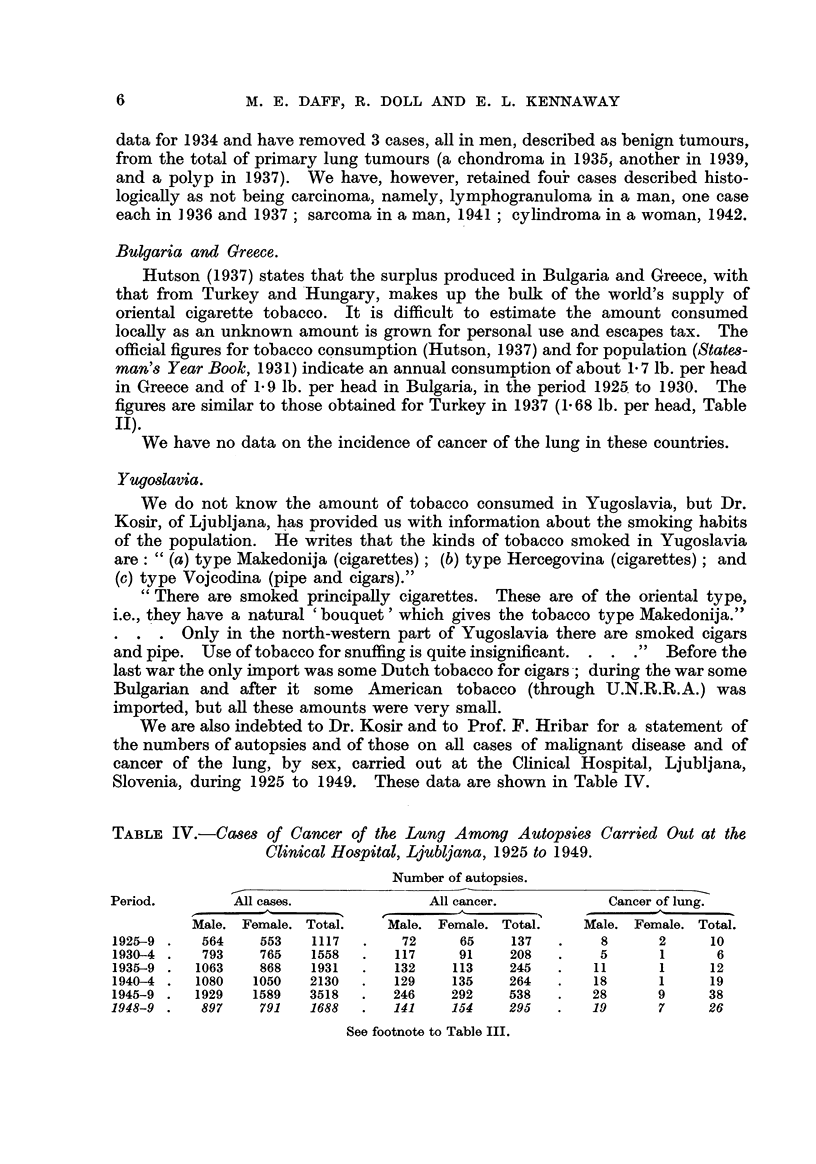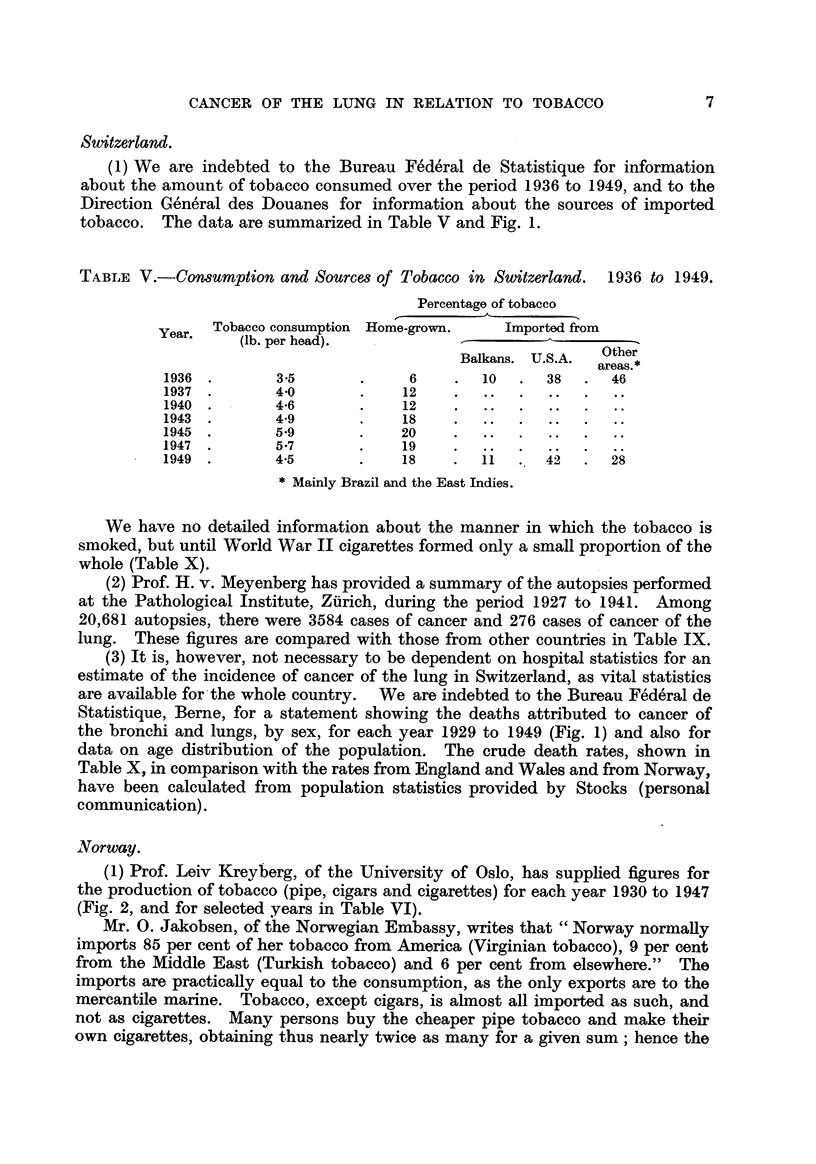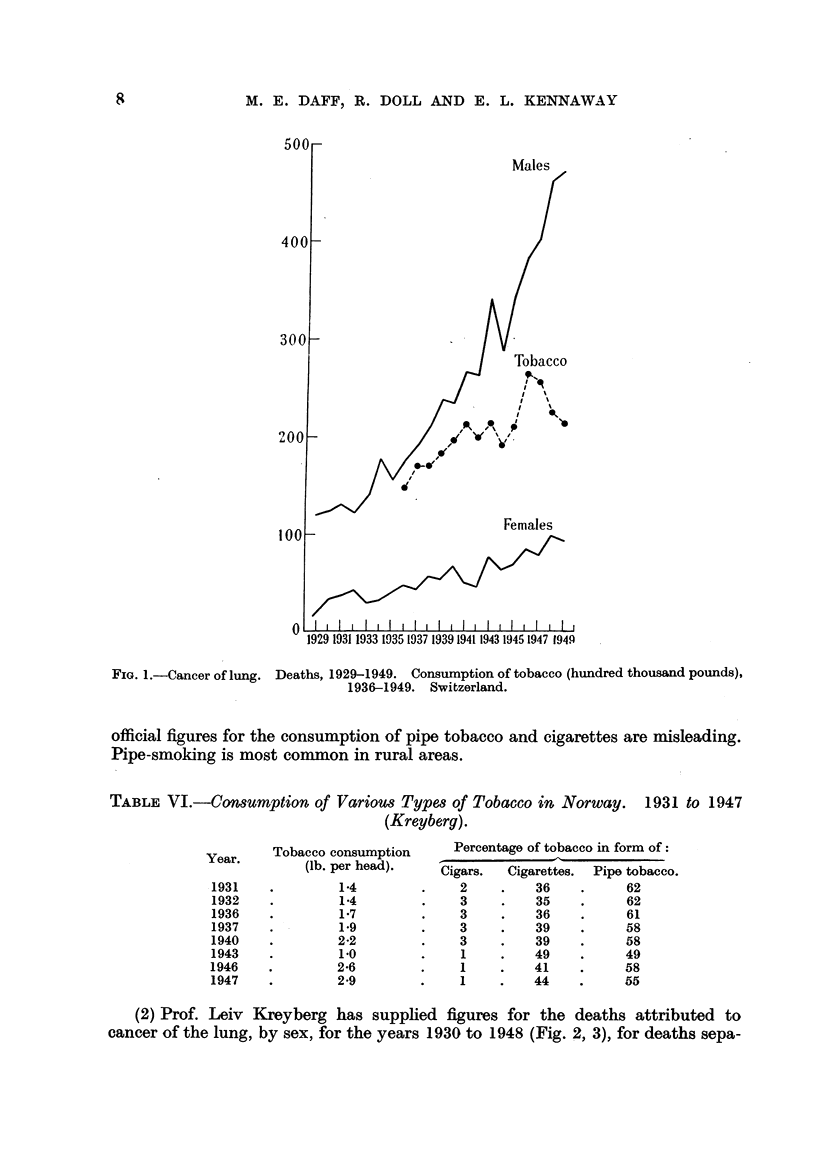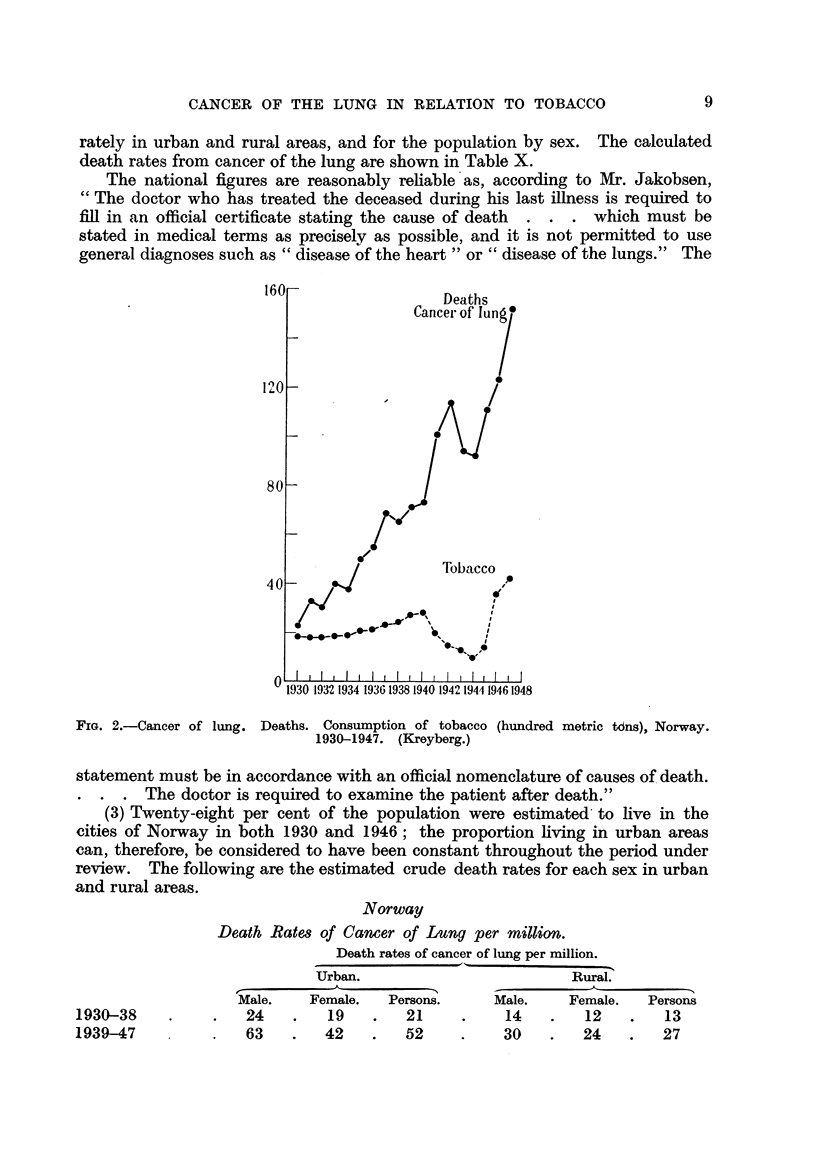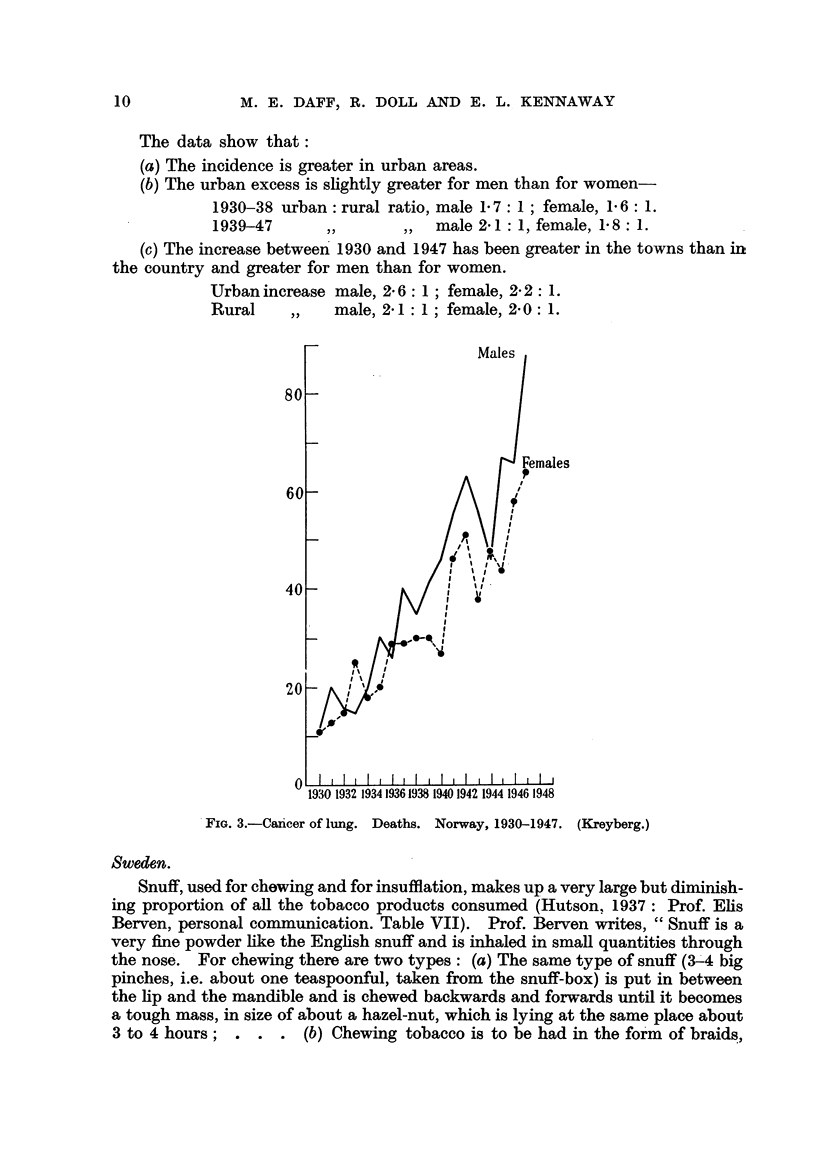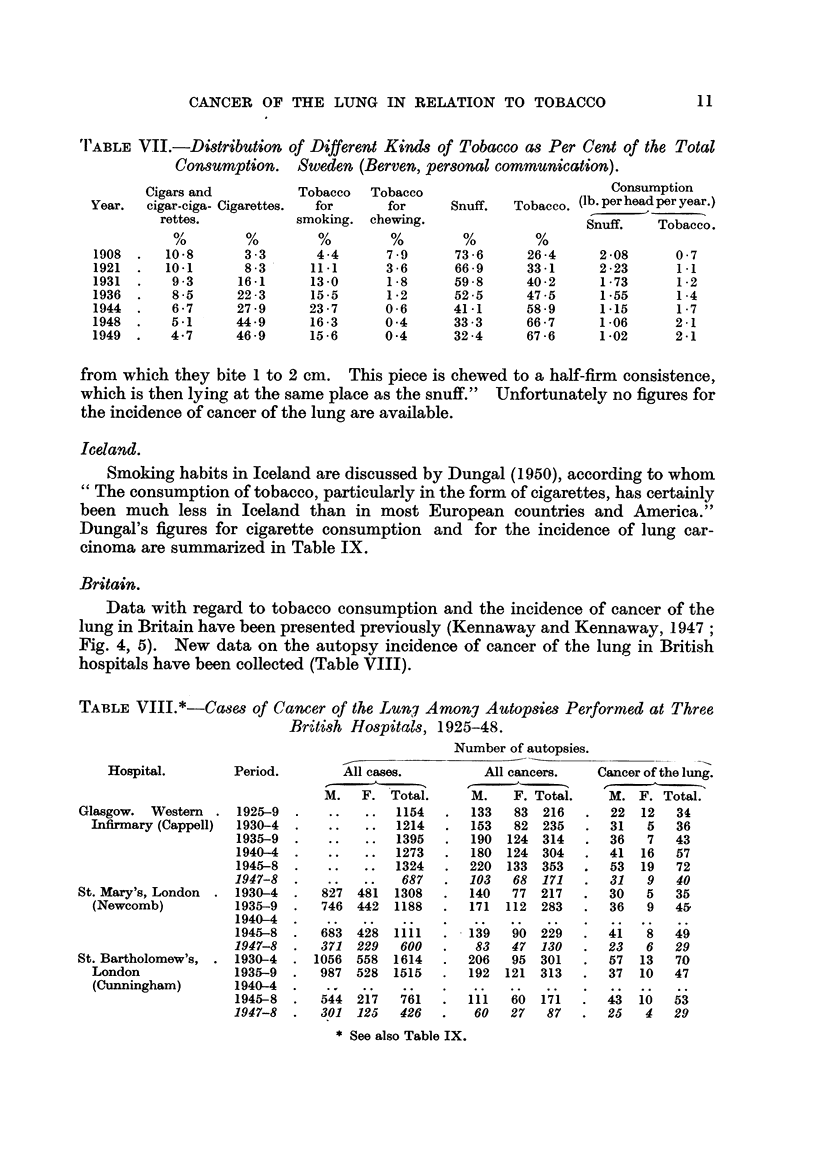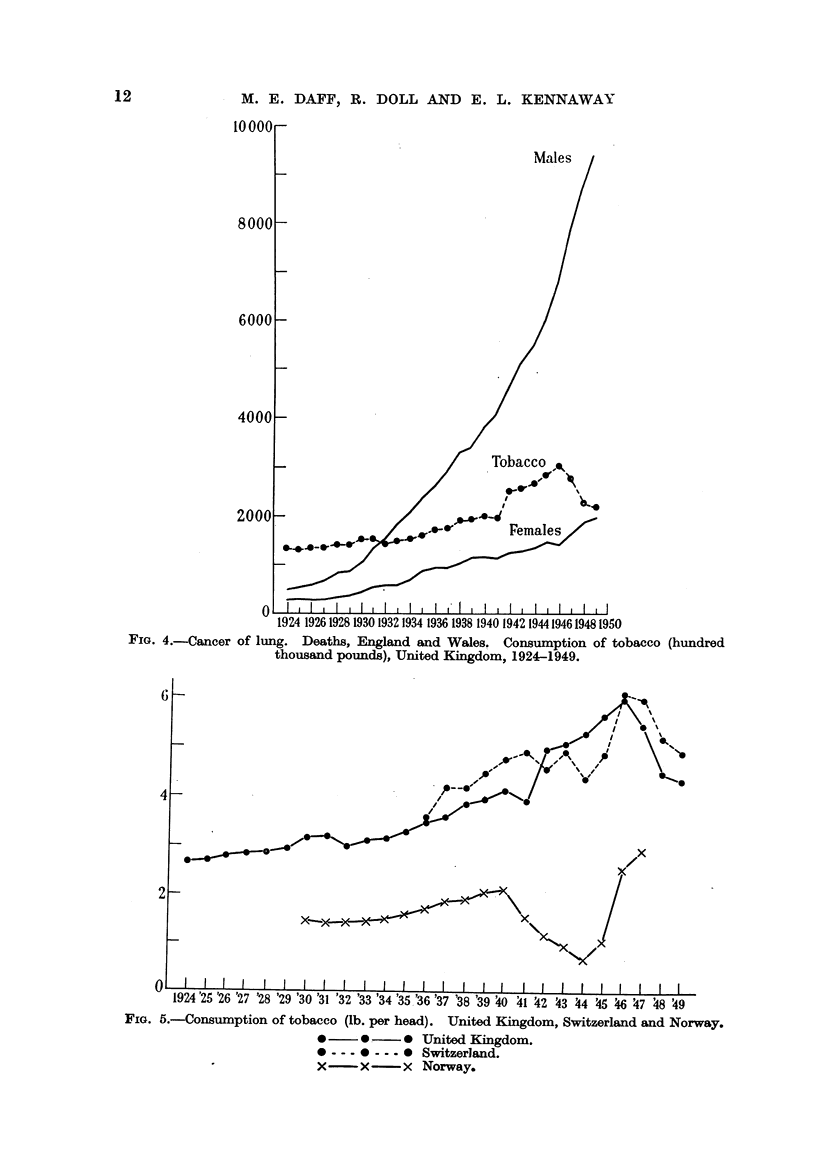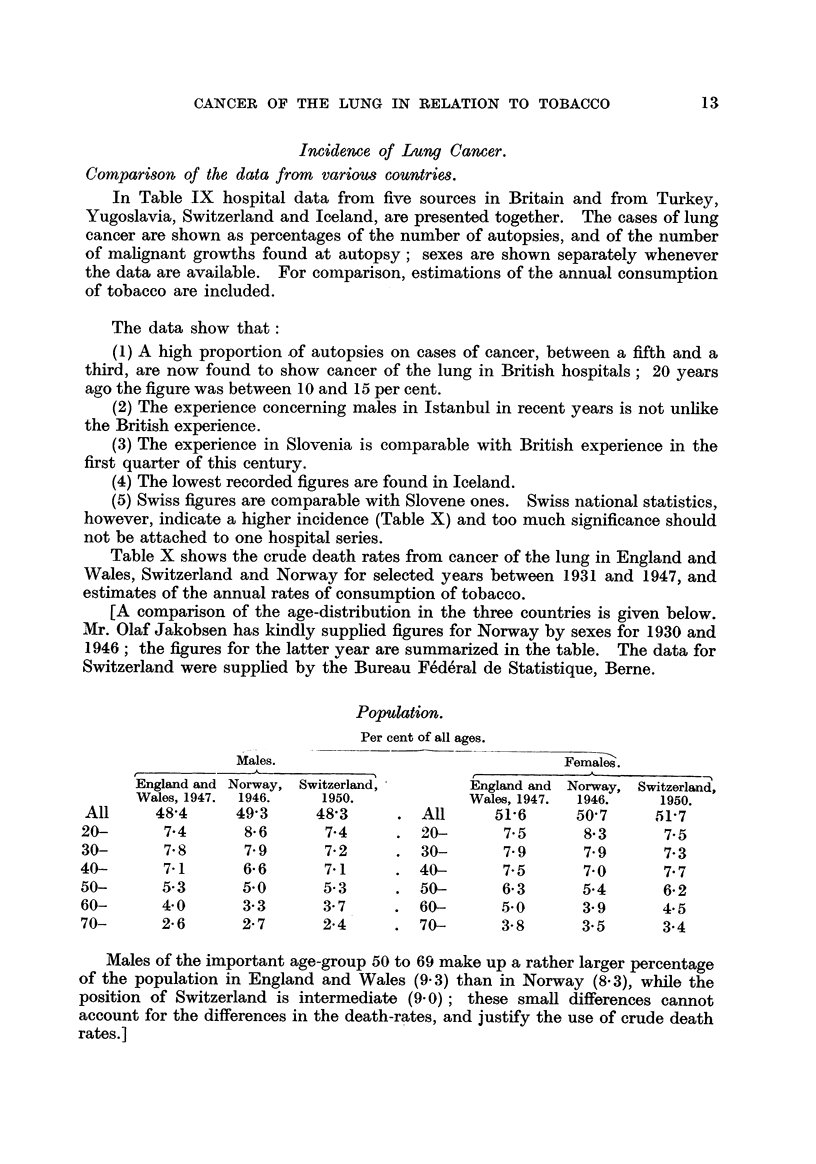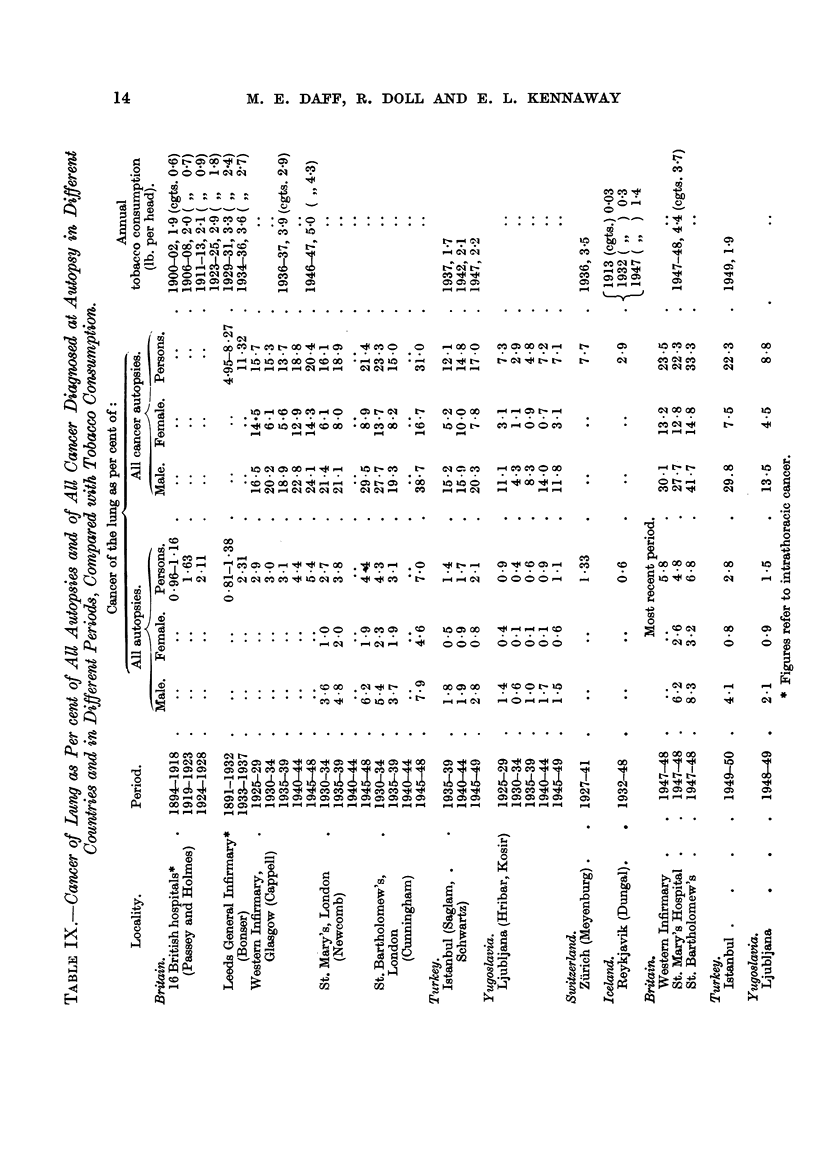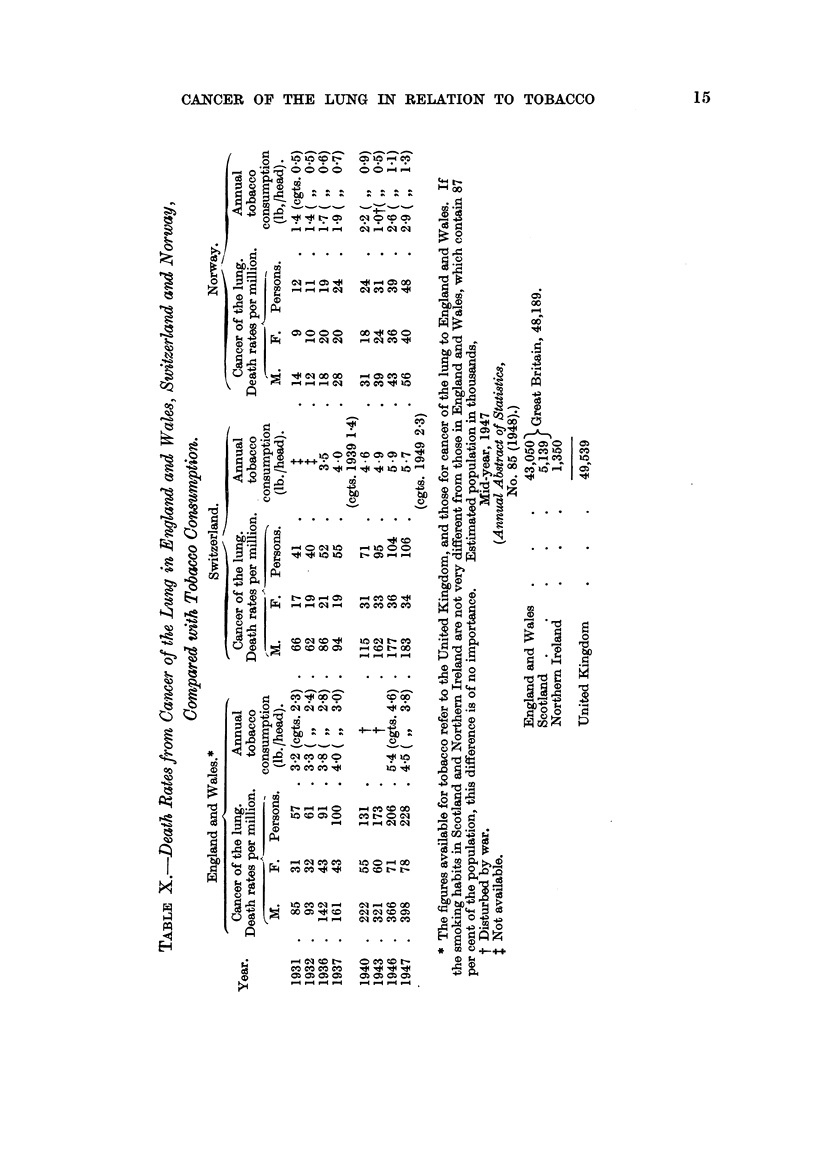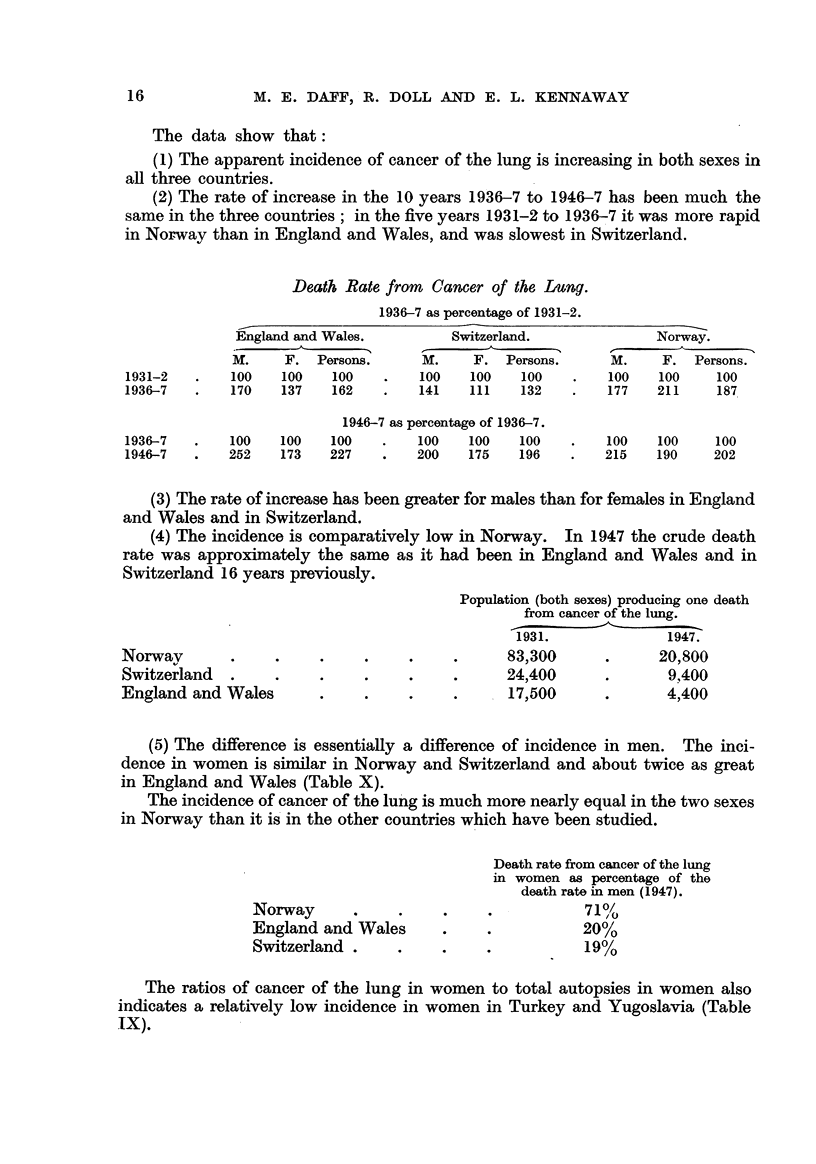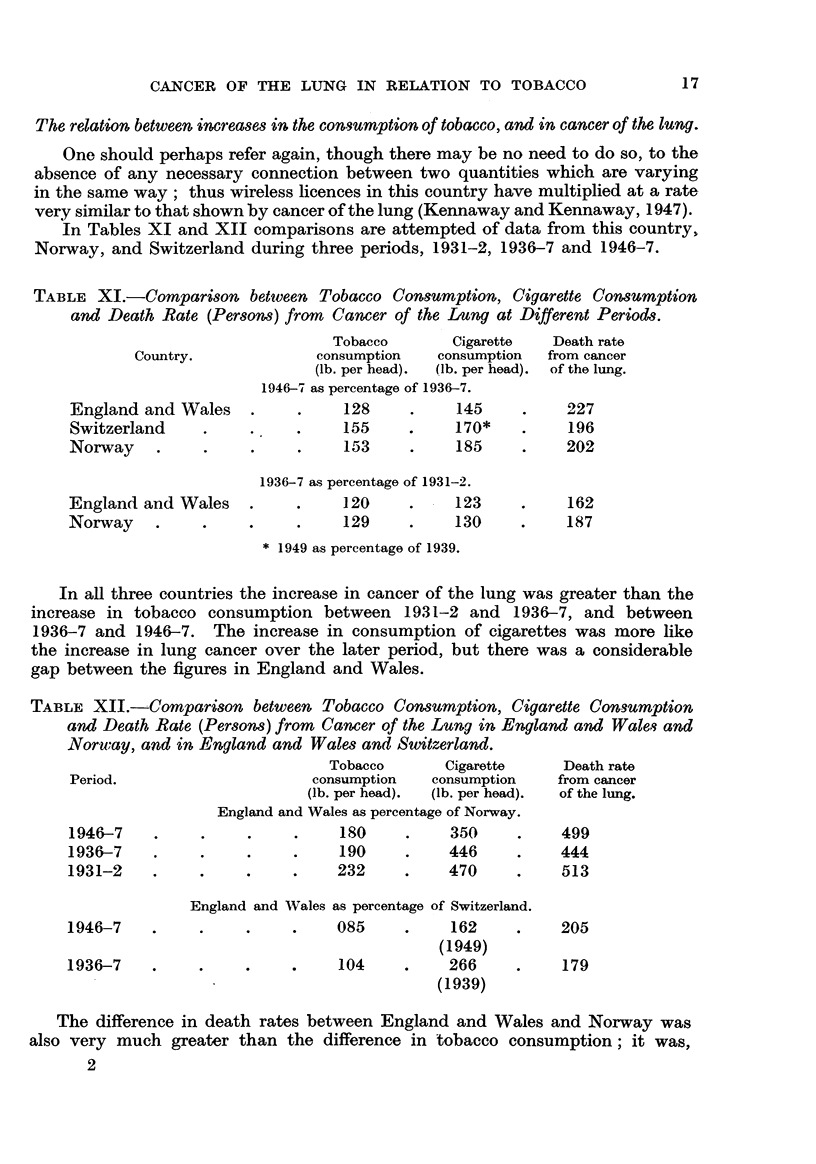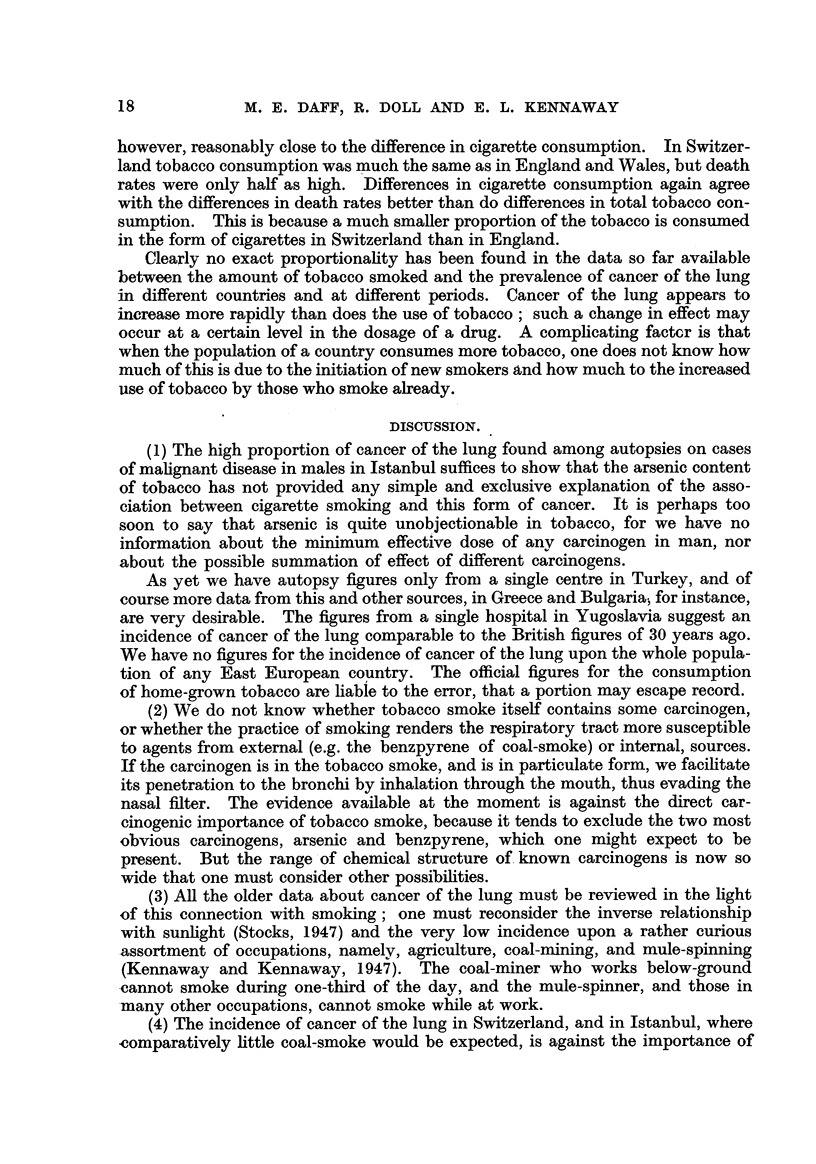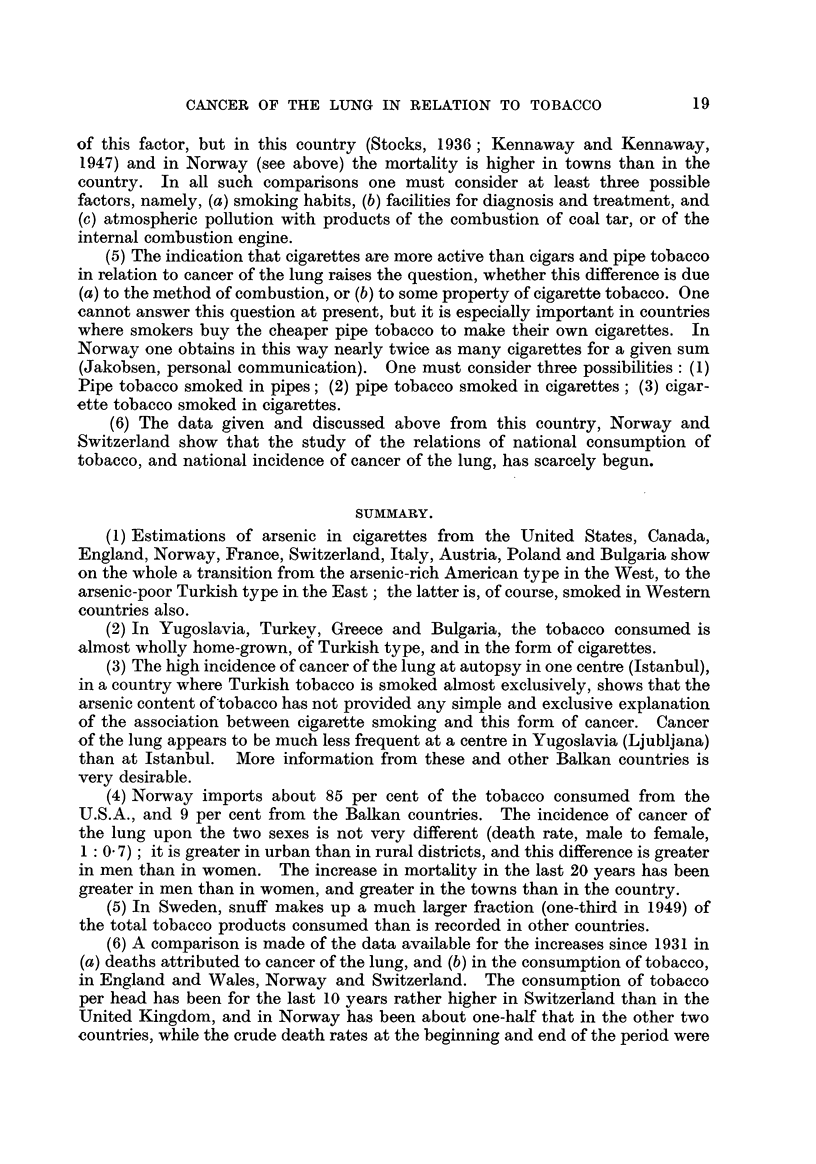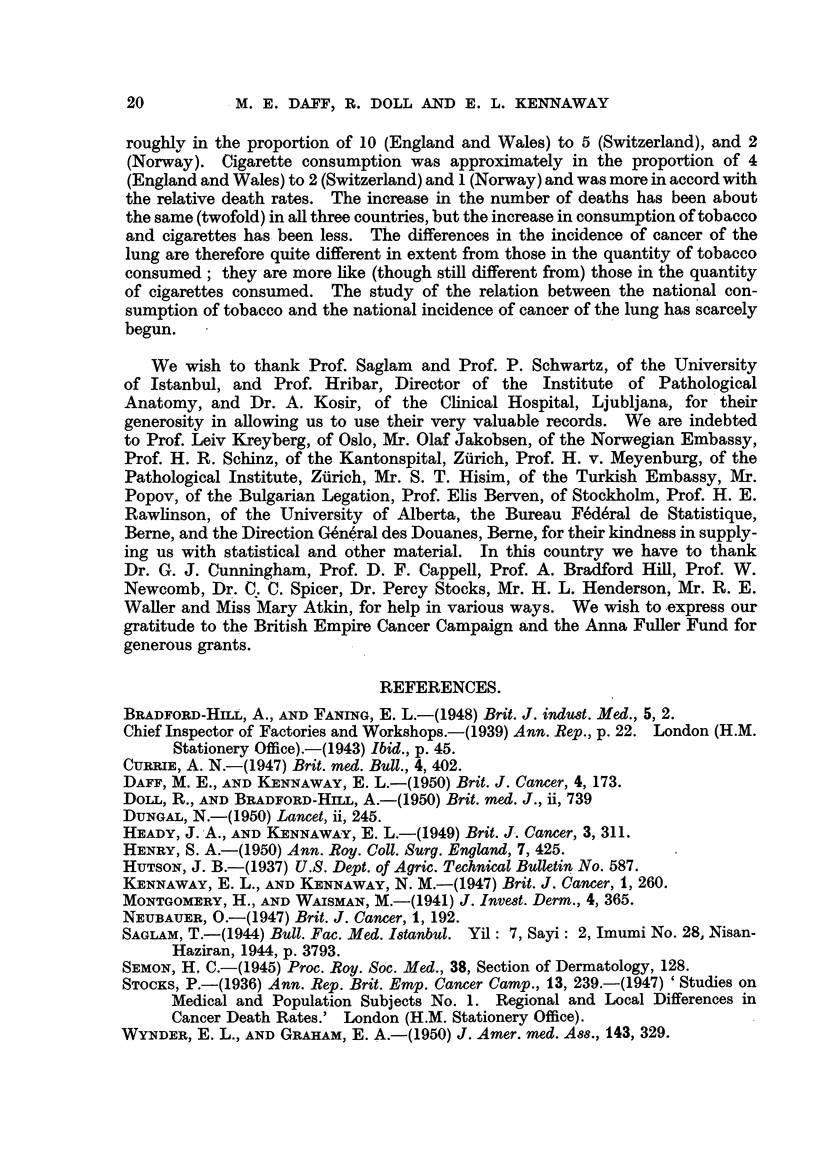# Cancer of the Lung in Relation to Tobacco

**DOI:** 10.1038/bjc.1951.1

**Published:** 1951-03

**Authors:** M. E. Daff, R. Doll, E. L. Kennaway


VOL. V MARCH, 1951 NO. 1

### CANCER OF THE LUNG IN RELATION TO TOBACCO

M. E. DAFF, R. DOLL AND E. L. KENNAWAY.

*From the Pathological Department, St. Bartholomew's Hospital, and the Statistical Research Unit of the Medical Research Council, London School of Hygiene and Tropical Medicine.*

Received for publication February 20, 1951

### *Further Data on the Arsenic Content of Cigarettes.*

In an earlier paper (Daff and Kennaway, 1950) data were given for the arsenic content of 5 brands of cigarettes of British and American types, of 8 brands of Turkish type, and of two others (French, Rhodesian), and also for the amount of arsenic volatilized in smoking. A further series of estimations of arsenic in cigarettes from 8 countries (U.S.A., Canada, Norway, Switzerland, Austria, Italy, Bulgaria, Poland) is reported in Table I. In all, 27 brands have been examined.

All quantities of arsenic given in this paper are stated as $As_2O_3$ $\mu g/g.$, which is numerically the same as parts per million.

The results given in this and the earlier paper show—

(1) A high arsenic content (24 to 106) in 10 non-Turkish brands smoked in England, U.S.A., Canada and Norway.

(2) A low arsenic content (0·0 to 4·3) in 8 Turkish brands as sold in England, in a Rhodesian brand, in a popular Austrian brand, and in cigarettes from France, Poland and Bulgaria.

(3) Intermediate amounts (3·1 to 12·0) in a less popular Austrian brand, and in cigarettes from Switzerland and Italy. One Canadian brand showed a range of 8·6 to 18·7.

Of course no claim is made that the analyses of these cigarettes, which we have happened to obtain through the kindness of various friends, can give any complete picture of the comparative habits of smokers in the different countries. But most of the brands are popular ones and the results show, on the whole, a gradation from the arsenic-rich American type in Western Europe to the arsenic-poor Turkish type in the East, with an intermediate mid-European zone, which accords with Hutson's (1937) account of tobacco culture in Europe quoted below (p. 3).

### *Cancer of the Lung in Relation to the Types of Tobacco Smoked in Various Countries.*

Some recent investigations, and especially those of Wynder and Graham (1950), and of Doll and Bradford Hill (1950), show an association between the smoking of tobacco, and especially of cigarettes, and cancer of the lung. Since arsenic is under

TABLE I.—*Arsenic Content of Cigarettes. Brands other than English.*

| Brand. | Number of cigarettes. | $As_2O_3$ μg. per g. |
|---|---|---|
| *U.S.A.* | | |
| Brand C | 2 | 46·4 ; 46·7. |
| „ P.M. | 1 | 51·0. |
| *Canada.* | | |
| Brand K | 4 | 36·8 ; 58·2 ; 47·1 ; 55·1. |
| „ O | 5 | 8·7 ; 18·4 ; 8·6 ; 18·7 ; 9·1. |
| „ W | 6 | 41·1 ; 46·5 ; 57·9 ; 79·5 ; 67·5 ; 41·7. |
| „ B.C. | 3 | 43·5 ; 57·6 ; 42·5. |
| *Norway* | | |
| Brand B | 3 | 71·7 ; 65·8 ; 74·6. |
| „ T | 4 | 54·4 ; 52·4 ; 55·6 ; 49·7. |
| *Switzerland.* | | |
| Brand P | 4 | 6·4 ; 3·1 ; 3·9 ; 3·4. |
| *Italy.* | | |
| Brand N | 6 | 10·5 ; 9·8 ; 10·2 ; 13·7 ; 9·1 ; 8·3. |
| *Austria.* | | |
| Brand D | 1 | 12·0. |
| „ M | 3 | Trace ; 2·1 ; 0·9. |
| *Poland.* | | |
| Brand Z | 5 | 2·5 ; 2·1 ; 1·2 ; 2·7 ; 1·5. |
| *Bulgaria.* | | |
| Brand A | 4 | 0·7 ; 1·2 ; nil ; 0·3. |
| „ R | 4 | 0·6 ; 0·6 ; nil ; 0·2. |

some circumstances carcinogenic in man (Neubauer, 1947 ; Currie, 1947 ; Bradford Hill and Faning, 1948), the question arises, whether the arsenic in tobacco is concerned in this process. Instances of cancer of the lung in workers exposed to arsenic are given by the Chief Inspector of Factories (1939). In a case of arsenical poisoning " . . . due to sodium arsenite, which was fatal, the post-mortem examination revealed that in addition to pigmentation of the trunk and limbs, warty growths all over the body and perforation of the nasal septum, there was a primary cancer of the right lung with metastatic growths in neighbouring glands and in the liver." In a later Report (Chief Inspector of Factories, 1943) another case is recorded " . . . in a filling machine operator, aged 57, for 43 years in a factory manufacturing sheep dip containing sodium arsenite, the cause of death being due to carcinoma of the right lung. Three similar cases of pulmonary carcinoma occurring in arsenical sheep-dip workers have been notified since 1939." Henry (1950) describes a case of cutaneous cancer " . . . in a furnaceman making sodium arsenite who eventually died of cancer of the lung . . ." Two non-industrial cases of bronchial cancer are recorded in persons taking arsenic medicinally over long periods (Montgomery and Waisman, 1941 ; Semon, 1945). This question might be decided if one could have equally reliable statistics for the incidence of cancer of the lung in countries where the predominant tobacco smoked is of the arsenic-rich, or of the arsenic-poor, type. We have been collecting data on this question by correspondence, but this is a slow and difficult process. In view of the great practical importance of the matter it seems better to publish the available data now in the hope of promoting first-hand inquiry in appropriate countries.

So far as we know, the only statistical studies of cancer of the lung in relation to cigarette smoking which have come from any countries other than Great Britain, the U.S.A. and Germany are those of Saglam (1944) from Turkey and of Dungal (1950) from Iceland.

The data which one would like to obtain from any given country are :

A. *From national sources.*—(1) Total consumption of tobacco. (2) Proportion of tobacco which is of American, or Turkish, type. (3) Proportion of tobacco smoked as cigarettes, cigars and pipe-tobacco. (4) Population by ages. (5) Number of deaths attributed to cancer of the lung. The two last items for men and women separately.

B. *From university clinics* (for men and women separately—preferably by ages).—(1) Number of autopsies. (2) Number of autopsies showing cancer. (3) Number of autopsies showing cancer of lung.

In the countries where A(5) is not available one must rely on B. The ratio of B(3) to B(1) has been suggested as the most reliable index for the present purpose (Heady and Kennaway, 1949). Figures for a number of separate years are, of course, very desirable, but those for the war period 1940 to 1945 generally show considerable disturbances (Fig. 2, 5) and must be avoided. We are indebted to the staffs of various Embassies in London for data on A(1), (2), (3) and (4), in addition to what can be found in such works of reference as the *Statesman's Year Book.* A great deal of information is given in a report, " The Consumption and Production of Tobacco in Europe," by J. B. Hutson (1937).

In such comparisons between different countries at least four possible carcinogenic factors must be considered, namely : (1) Smoking of tobacco. (2) Smoking of tobacco containing arsenic. (3) Inhalation of the dust of towns and of coal smoke. (4) Inhalation of products of the internal combustion engine.

Thus populations in eastern Europe which are subject to the first of these factors, and only slightly if at all to the second, will also be comparatively free from the third and fourth, while the industrial peoples of Britain and the U.S.A. will be most exposed to the second, third and fourth together, and the inhabitants of Iceland will be subject to the second only.

### *Tobacco Culture in Europe.*

Hutson (1937) classifies the types of tobacco grown in Europe thus :

(1) Oriental : Turkey, Greece, Bulgaria, S. Yugoslavia, U.S.S.R.

(2) Semi-Oriental : S.E. Italy, S.W. Yugoslavia, Rumania, Hungary, S. Czechoslovakia, S. Poland.

(3) Dark air-cured : Hungary, Rumania, Czechoslovakia, Germany, N. Italy, France, Spain, Belgium, Sweden, Switzerland.

He divides the countries of Europe into 4 groups :

(1) Those in which a portion of the factory consumption is grown (Germany, Spain, Belgium, Poland, Czechoslovakia, Switzerland).

(2) Those in which production is approximately equal to consumption (Italy, Yugoslavia, Rumania).

(3) Those which produce a surplus (Greece, Turkey, Bulgaria, Hungary).

(4) Those in which little or no tobacco is grown, which are all those not named above.

The countries in the third group are of most interest, if one can assume that the tobacco smoked there is at any rate very largely of the arsenic-poor type, but unfortunately the conditions for the study of cancer of the lung in the whole population are not as yet very favourable in them, and this subject, like that of cancer of the liver, presents peculiar difficulties on account of the danger of confusion of primary and secondary growths.

*Tobacco Consumption and Cancer of the Lung in Various Countries.*
*Turkey.*

(1) Data on the consumption of tobacco in Turkey are given in Table II.

TABLE II.—*Consumption of Tobacco in Turkey.*

| Year. | Tobacco consumption (million lb.) | Population. | Tobacco consumption (lb. per head.) |
|---|---|---|---|
| 1925 | 15·8* | 13,023,000 | 1·21 |
| 1930 | 21·9* | 14,591,000 | 1·50 |
| 1935 | 25·0* | 16,158,018 | 1·55 |
| 1937 | 28·2† | 16,800,000 | 1·68 |
| 1942 | 37·2† | 17,820,950 | 2·09 |
| 1947 | 40·7† | 18,870,785 | 2·16 |
| 1949 | 39·7† | 20,900,009‡ | 1·90 |

* Figures from Hutson (1937).

† Figures provided by the Turkish Embassy.

‡ *The Times*, October 30, 1950.

(2) Prof. Schwartz, of the University of Istanbul, writes that in Turkey tobacco is consumed almost wholly in the form of cigarettes ; that cigars, European pipes and the oriental water-pipe are almost unknown among the bulk of the people, and that there are many heavy smokers among women.

(3) The only publication from any Balkan country upon cancer of the lung in relation to smoking appears to be that of Prof. Saglam (1944) of the University of Istanbul, who gives the following details, which are quoted in full in view of the importance of his original paper, and the difficulty of access to it.

" The clinical statistics of pulmonary carcinoma in Turkey :

| | Year. | Number of patients. | Pulmonary tumour. | Rate per thousand. |
|---|---|---|---|---|
| The Int. Clinic of Gulhane (Suleyman Numan) | 1899–1908 | 4270 | 2 | 0·46 (0·47)* |
| The Int. Clinic of Gulhane (Tevfik Saglam) | 1923–1926 | 2343 | 7 | 2·1 (3·0)* |
| The Sect. for Int. Diseases of Gureba (Tevfik Saglam) | 1931–32 | 1459 | 9 | 7·1 (6·2)* |
| Haydarpaxa Numune Hast. (Tevfik Saglam) | 1936–1939 | 4230 | 24 | 5·6 (5·7)* |

* Recalculated from the figures given by Prof. Saglam in the two adjacent columns.

" The anatomo-pathologic statistics :

| | Year. | Number of autopsies. | Pulmonary cancer. | Rate per thousand. |
|---|---|---|---|---|
| Gulhane (Deycke-Reinhard) | 1899–1908 | 800 | 3 | 3·7 |
| Gulhane (Lufti) | 1923–1926 | 502 | 5 | 10 |
| Haydarpasa Numune Hast. | 1936–1939 | 635 | 14 | 22 |
| The Anatomo-pathologic Institute of the University of Istanbul (Prof. Ph. Schwartz) | 1935–1943 | 5126 | 79 | 15·4 |

" Taking the clinical statistics into consideration it is clear that within 30–40 years the increase in our clinics is 12 times and according to the anatomo-pathologic statistics it is 4·1 times as much."

" We have seen 11 cases of pulmonary carcinoma in the newly inaugurated 3. Ic Hastaliklari Klinigi in 23 months (among 2084 patients, 5·3 in a thousand)."

" All these statistics show that in our country, within the last 30 years, pulmonary carcinoma has increased in a great proportion."

Saglam rejects cigarette-smoking as a factor in the incidence of bronchial carcinoma. " Some authors believe that there exists a relationship between smoking tobacco and inhaling the smoke and pulmonary carcinoma. Animal experiments seem to support this point of view. Some authors are of the opinion that especially cigarettes play a great part in its production. We do not hold the same view. For a long time, at least since 50 years, almost only cigarettes are smoked in our country. In spite of this the increase in pulmonary Ca. in this country has gone on the same pace as in those countries where cigars and pipes are mostly smoked. The amount of tobacco consumed in our country has shown a constant increase, and from 9,370,000 kgr. in 1926 has risen to 16,680,000 kgr. in 1942. But this rise is not sufficient to explain the increase in pulmonary Ca."

But there is no exact basis for any such argument. We do not yet know what consumption of any tobacco produces any given incidence of bronchial carcinoma, nor do we know the time-relations of any such effect.

(4) We are indebted to Prof. Saglam, Prof. Schwartz and Dr. Yenermen for (*a*) full protocols of the 193 autopsies on cases of primary tumour of the lung carried out at the University of Istanbul during 1934–1950, and (*b*) figures for the total autopsies and autopsies on cases of cancer, by sexes, for the same period (Table III).

To make the material comparable with other series, we have omitted the

TABLE III.—*Cases of Cancer of the Lung Among Autopsies Carried Out at the University of Istanbul,* 1935–1950.

| Period. | Number of autopsies. All cases. Male. | Female. | Total. | All cancer. Male. | Female. | Total. | Cancer of lung. Male. | Female. | Total. |
|---|---|---|---|---|---|---|---|---|---|
| 1935–9 | 1833 | 947 | 2780 | 217 | 97 | 314 | 33 | 5 | 38 |
| 1940–4 | 2585 | 747 | 3332 | 302 | 70 | 372 | 48 | 7 | 55 |
| 1945–9 | 2612 | 1323 | 3935 | 359 | 129 | 488 | 73 | 10 | 83 |
| *1949–50* | *767* | *488* | *1255* | *104* | *53* | *157* | *31* | *4* | *35* |

Changes in incidence will be seen more clearly in Table IX, where cases of cancer of the lung are shown as percentages of all autopsies and of all cancers. Note that data for the year 1949 appear twice.

data for 1934 and have removed 3 cases, all in men, described as benign tumours, from the total of primary lung tumours (a chondroma in 1935, another in 1939, and a polyp in 1937). We have, however, retained four cases described histologically as not being carcinoma, namely, lymphogranuloma in a man, one case each in 1936 and 1937 ; sarcoma in a man, 1941 ; cylindroma in a woman, 1942.

*Bulgaria and Greece.*

Hutson (1937) states that the surplus produced in Bulgaria and Greece, with that from Turkey and Hungary, makes up the bulk of the world's supply of oriental cigarette tobacco. It is difficult to estimate the amount consumed locally as an unknown amount is grown for personal use and escapes tax. The official figures for tobacco consumption (Hutson, 1937) and for population (*Statesman's Year Book*, 1931) indicate an annual consumption of about 1·7 lb. per head in Greece and of 1·9 lb. per head in Bulgaria, in the period 1925 to 1930. The figures are similar to those obtained for Turkey in 1937 (1·68 lb. per head, Table II).

We have no data on the incidence of cancer of the lung in these countries.

*Yugoslavia.*

We do not know the amount of tobacco consumed in Yugoslavia, but Dr. Kosir, of Ljubljana, has provided us with information about the smoking habits of the population. He writes that the kinds of tobacco smoked in Yugoslavia are : " (*a*) type Makedonija (cigarettes) ; (*b*) type Hercegovina (cigarettes) ; and (*c*) type Vojcodina (pipe and cigars)."

" There are smoked principally cigarettes. These are of the oriental type, i.e., they have a natural ' bouquet ' which gives the tobacco type Makedonija." . . . Only in the north-western part of Yugoslavia there are smoked cigars and pipe. Use of tobacco for snuffing is quite insignificant. . . ." Before the last war the only import was some Dutch tobacco for cigars ; during the war some Bulgarian and after it some American tobacco (through U.N.R.R.A.) was imported, but all these amounts were very small.

We are also indebted to Dr. Kosir and to Prof. F. Hribar for a statement of the numbers of autopsies and of those on all cases of malignant disease and of cancer of the lung, by sex, carried out at the Clinical Hospital, Ljubljana, Slovenia, during 1925 to 1949. These data are shown in Table IV.

TABLE IV.—*Cases of Cancer of the Lung Among Autopsies Carried Out at the Clinical Hospital, Ljubljana,* 1925 *to* 1949.

| Period. | Number of autopsies. All cases. | | | All cancer. | | | Cancer of lung. | | |
|---|---|---|---|---|---|---|---|---|---|
| | Male. | Female. | Total. | Male. | Female. | Total. | Male. | Female. | Total. |
| 1925–9 | 564 | 553 | 1117 | 72 | 65 | 137 | 8 | 2 | 10 |
| 1930–4 | 793 | 765 | 1558 | 117 | 91 | 208 | 5 | 1 | 6 |
| 1935–9 | 1063 | 868 | 1931 | 132 | 113 | 245 | 11 | 1 | 12 |
| 1940–4 | 1080 | 1050 | 2130 | 129 | 135 | 264 | 18 | 1 | 19 |
| 1945–9 | 1929 | 1589 | 3518 | 246 | 292 | 538 | 28 | 9 | 38 |
| *1948–9* | *897* | *791* | *1688* | *141* | *154* | *295* | *19* | *7* | *26* |

See footnote to Table III.

*Switzerland.*

(1) We are indebted to the Bureau Fédéral de Statistique for information about the amount of tobacco consumed over the period 1936 to 1949, and to the Direction Général des Douanes for information about the sources of imported tobacco. The data are summarized in Table V and Fig. 1.

TABLE V.—*Consumption and Sources of Tobacco in Switzerland.* 1936 *to* 1949.

| Year. | Tobacco consumption (lb. per head). | Percentage of tobacco: Home-grown. | Imported from: Balkans. | U.S.A. | Other areas.* |
|---|---|---|---|---|---|
| 1936 | 3·5 | 6 | 10 | 38 | 46 |
| 1937 | 4·0 | 12 | .. | .. | .. |
| 1940 | 4·6 | 12 | .. | .. | .. |
| 1943 | 4·9 | 18 | .. | .. | .. |
| 1945 | 5·9 | 20 | .. | .. | .. |
| 1947 | 5·7 | 19 | .. | .. | .. |
| 1949 | 4·5 | 18 | 11 | 42 | 28 |

* Mainly Brazil and the East Indies.

We have no detailed information about the manner in which the tobacco is smoked, but until World War II cigarettes formed only a small proportion of the whole (Table X).

(2) Prof. H. v. Meyenberg has provided a summary of the autopsies performed at the Pathological Institute, Zürich, during the period 1927 to 1941. Among 20,681 autopsies, there were 3584 cases of cancer and 276 cases of cancer of the lung. These figures are compared with those from other countries in Table IX.

(3) It is, however, not necessary to be dependent on hospital statistics for an estimate of the incidence of cancer of the lung in Switzerland, as vital statistics are available for the whole country. We are indebted to the Bureau Fédéral de Statistique, Berne, for a statement showing the deaths attributed to cancer of the bronchi and lungs, by sex, for each year 1929 to 1949 (Fig. 1) and also for data on age distribution of the population. The crude death rates, shown in Table X, in comparison with the rates from England and Wales and from Norway, have been calculated from population statistics provided by Stocks (personal communication).

*Norway.*

(1) Prof. Leiv Kreyberg, of the University of Oslo, has supplied figures for the production of tobacco (pipe, cigars and cigarettes) for each year 1930 to 1947 (Fig. 2, and for selected years in Table VI).

Mr. O. Jakobsen, of the Norwegian Embassy, writes that " Norway normally imports 85 per cent of her tobacco from America (Virginian tobacco), 9 per cent from the Middle East (Turkish tobacco) and 6 per cent from elsewhere." The imports are practically equal to the consumption, as the only exports are to the mercantile marine. Tobacco, except cigars, is almost all imported as such, and not as cigarettes. Many persons buy the cheaper pipe tobacco and make their own cigarettes, obtaining thus nearly twice as many for a given sum ; hence the

FIG. 1.—Cancer of lung. Deaths, 1929–1949. Consumption of tobacco (hundred thousand pounds), 1936–1949. Switzerland.

official figures for the consumption of pipe tobacco and cigarettes are misleading. Pipe-smoking is most common in rural areas.

TABLE VI.—*Consumption of Various Types of Tobacco in Norway.* 1931 *to* 1947 (*Kreyberg*).

| Year. | Tobacco consumption (lb. per head). | Percentage of tobacco in form of : | | |
|---|---|---|---|---|
| | | Cigars. | Cigarettes. | Pipe tobacco. |
| 1931 | 1·4 | 2 | 36 | 62 |
| 1932 | 1·4 | 3 | 35 | 62 |
| 1936 | 1·7 | 3 | 36 | 61 |
| 1937 | 1·9 | 3 | 39 | 58 |
| 1940 | 2·2 | 3 | 39 | 58 |
| 1943 | 1·0 | 1 | 49 | 49 |
| 1946 | 2·6 | 1 | 41 | 58 |
| 1947 | 2·9 | 1 | 44 | 55 |

(2) Prof. Leiv Kreyberg has supplied figures for the deaths attributed to cancer of the lung, by sex, for the years 1930 to 1948 (Fig. 2, 3), for deaths separately in urban and rural areas, and for the population by sex. The calculated death rates from cancer of the lung are shown in Table X.

The national figures are reasonably reliable as, according to Mr. Jakobsen, "The doctor who has treated the deceased during his last illness is required to fill in an official certificate stating the cause of death . . . which must be stated in medical terms as precisely as possible, and it is not permitted to use general diagnoses such as "disease of the heart" or "disease of the lungs." The

Fig. 2.—Cancer of lung. Deaths. Consumption of tobacco (hundred metric tons), Norway. 1930–1947. (Kreyberg.)

statement must be in accordance with an official nomenclature of causes of death. . . . The doctor is required to examine the patient after death."

(3) Twenty-eight per cent of the population were estimated to live in the cities of Norway in both 1930 and 1946; the proportion living in urban areas can, therefore, be considered to have been constant throughout the period under review. The following are the estimated crude death rates for each sex in urban and rural areas.

*Norway*

*Death Rates of Cancer of Lung per million.*

| | Death rates of cancer of lung per million. | | | | | |
|---|---|---|---|---|---|---|
| | Urban. | | | Rural. | | |
| | Male. | Female. | Persons. | Male. | Female. | Persons |
| 1930–38 | 24 | 19 | 21 | 14 | 12 | 13 |
| 1939–47 | 63 | 42 | 52 | 30 | 24 | 27 |

The data show that :

(*a*) The incidence is greater in urban areas.

(*b*) The urban excess is slightly greater for men than for women—

1930–38 urban : rural ratio, male 1·7 : 1 ; female, 1·6 : 1.
1939–47 ,, ,, male 2·1 : 1, female, 1·8 : 1.

(*c*) The increase between 1930 and 1947 has been greater in the towns than in the country and greater for men than for women.

Urban increase male, 2·6 : 1 ; female, 2·2 : 1.
Rural ,, male, 2·1 : 1 ; female, 2·0 : 1.

FIG. 3.—Cancer of lung. Deaths. Norway, 1930–1947. (Kreyberg.)

*Sweden.*

Snuff, used for chewing and for insufflation, makes up a very large but diminishing proportion of all the tobacco products consumed (Hutson, 1937 : Prof. Elis Berven, personal communication. Table VII). Prof. Berven writes, " Snuff is a very fine powder like the English snuff and is inhaled in small quantities through the nose. For chewing there are two types : (*a*) The same type of snuff (3–4 big pinches, i.e. about one teaspoonful, taken from the snuff-box) is put in between the lip and the mandible and is chewed backwards and forwards until it becomes a tough mass, in size of about a hazel-nut, which is lying at the same place about 3 to 4 hours ; . . . (*b*) Chewing tobacco is to be had in the form of braids,

TABLE VII.—*Distribution of Different Kinds of Tobacco as Per Cent of the Total Consumption. Sweden (Berven, personal communication).*

| Year. | Cigars and cigar-cigarettes. | Cigarettes. | Tobacco for smoking. | Tobacco for chewing. | Snuff. | Tobacco. | Consumption (lb. per head per year.) Snuff. | Tobacco. |
|---|---|---|---|---|---|---|---|---|
| | % | % | % | % | % | % | | |
| 1908 . | 10·8 | 3·3 | 4·4 | 7·9 | 73·6 | 26·4 | 2·08 | 0·7 |
| 1921 . | 10·1 | 8·3 | 11·1 | 3·6 | 66·9 | 33·1 | 2·23 | 1·1 |
| 1931 . | 9·3 | 16·1 | 13·0 | 1·8 | 59·8 | 40·2 | 1·73 | 1·2 |
| 1936 . | 8·5 | 22·3 | 15·5 | 1·2 | 52·5 | 47·5 | 1·55 | 1·4 |
| 1944 . | 6·7 | 27·9 | 23·7 | 0·6 | 41·1 | 58·9 | 1·15 | 1·7 |
| 1948 . | 5·1 | 44·9 | 16·3 | 0·4 | 33·3 | 66·7 | 1·06 | 2·1 |
| 1949 . | 4·7 | 46·9 | 15·6 | 0·4 | 32·4 | 67·6 | 1·02 | 2·1 |

from which they bite 1 to 2 cm. This piece is chewed to a half-firm consistence, which is then lying at the same place as the snuff." Unfortunately no figures for the incidence of cancer of the lung are available.

*Iceland.*

Smoking habits in Iceland are discussed by Dungal (1950), according to whom "The consumption of tobacco, particularly in the form of cigarettes, has certainly been much less in Iceland than in most European countries and America." Dungal's figures for cigarette consumption and for the incidence of lung carcinoma are summarized in Table IX.

*Britain.*

Data with regard to tobacco consumption and the incidence of cancer of the lung in Britain have been presented previously (Kennaway and Kennaway, 1947 ; Fig. 4, 5). New data on the autopsy incidence of cancer of the lung in British hospitals have been collected (Table VIII).

TABLE VIII.*—*Cases of Cancer of the Lung Among Autopsies Performed at Three British Hospitals,* 1925–48.

| Hospital. | Period. | All cases. M. | All cases. F. | All cases. Total. | All cancers. M. | All cancers. F. | All cancers. Total. | Cancer of the lung. M. | Cancer of the lung. F. | Cancer of the lung. Total. |
|---|---|---|---|---|---|---|---|---|---|---|
| Glasgow. Western Infirmary (Cappell) | 1925–9 | .. | .. | 1154 | 133 | 83 | 216 | 22 | 12 | 34 |
| | 1930–4 | .. | .. | 1214 | 153 | 82 | 235 | 31 | 5 | 36 |
| | 1935–9 | .. | .. | 1395 | 190 | 124 | 314 | 36 | 7 | 43 |
| | 1940–4 | .. | .. | 1273 | 180 | 124 | 304 | 41 | 16 | 57 |
| | 1945–8 | .. | .. | 1324 | 220 | 133 | 353 | 53 | 19 | 72 |
| | *1947–8* | .. | .. | *687* | *103* | *68* | *171* | *31* | *9* | *40* |
| St. Mary's, London (Newcomb) | 1930–4 | 827 | 481 | 1308 | 140 | 77 | 217 | 30 | 5 | 35 |
| | 1935–9 | 746 | 442 | 1188 | 171 | 112 | 283 | 36 | 9 | 45 |
| | 1940–4 | .. | .. | .. | .. | .. | .. | .. | .. | .. |
| | 1945–8 | 683 | 428 | 1111 | 139 | 90 | 229 | 41 | 8 | 49 |
| | *1947–8* | *371* | *229* | *600* | *83* | *47* | *130* | *23* | *6* | *29* |
| St. Bartholomew's, London (Cunningham) | 1930–4 | 1056 | 558 | 1614 | 206 | 95 | 301 | 57 | 13 | 70 |
| | 1935–9 | 987 | 528 | 1515 | 192 | 121 | 313 | 37 | 10 | 47 |
| | 1940–4 | .. | .. | .. | .. | .. | .. | .. | .. | .. |
| | 1945–8 | 544 | 217 | 761 | 111 | 60 | 171 | 43 | 10 | 53 |
| | *1947–8* | *301* | *125* | *426* | *60* | *27* | *87* | *25* | *4* | *29* |

Number of autopsies.

* See also Table IX.

FIG. 4.—Cancer of lung. Deaths, England and Wales. Consumption of tobacco (hundred thousand pounds), United Kingdom, 1924–1949.

FIG. 5.—Consumption of tobacco (lb. per head). United Kingdom, Switzerland and Norway.

●——●——● United Kingdom.
● - - - ● - - - ● Switzerland.
×——×——× Norway.

### *Incidence of Lung Cancer.*

*Comparison of the data from various countries.*

In Table IX hospital data from five sources in Britain and from Turkey, Yugoslavia, Switzerland and Iceland, are presented together. The cases of lung cancer are shown as percentages of the number of autopsies, and of the number of malignant growths found at autopsy ; sexes are shown separately whenever the data are available. For comparison, estimations of the annual consumption of tobacco are included.

The data show that :

(1) A high proportion of autopsies on cases of cancer, between a fifth and a third, are now found to show cancer of the lung in British hospitals ; 20 years ago the figure was between 10 and 15 per cent.

(2) The experience concerning males in Istanbul in recent years is not unlike the British experience.

(3) The experience in Slovenia is comparable with British experience in the first quarter of this century.

(4) The lowest recorded figures are found in Iceland.

(5) Swiss figures are comparable with Slovene ones. Swiss national statistics, however, indicate a higher incidence (Table X) and too much significance should not be attached to one hospital series.

Table X shows the crude death rates from cancer of the lung in England and Wales, Switzerland and Norway for selected years between 1931 and 1947, and estimates of the annual rates of consumption of tobacco.

[A comparison of the age-distribution in the three countries is given below. Mr. Olaf Jakobsen has kindly supplied figures for Norway by sexes for 1930 and 1946 ; the figures for the latter year are summarized in the table. The data for Switzerland were supplied by the Bureau Fédéral de Statistique, Berne.

*Population.*

Per cent of all ages.

| | Males. | | | | Females. | | |
|---|---|---|---|---|---|---|---|
| | England and Wales, 1947. | Norway, 1946. | Switzerland, 1950. | | England and Wales, 1947. | Norway, 1946. | Switzerland, 1950. |
| All | 48·4 | 49·3 | 48·3 | All | 51·6 | 50·7 | 51·7 |
| 20– | 7·4 | 8·6 | 7·4 | 20– | 7·5 | 8·3 | 7·5 |
| 30– | 7·8 | 7·9 | 7·2 | 30– | 7·9 | 7·9 | 7·3 |
| 40– | 7·1 | 6·6 | 7·1 | 40– | 7·5 | 7·0 | 7·7 |
| 50– | 5·3 | 5·0 | 5·3 | 50– | 6·3 | 5·4 | 6·2 |
| 60– | 4·0 | 3·3 | 3·7 | 60– | 5·0 | 3·9 | 4·5 |
| 70– | 2·6 | 2·7 | 2·4 | 70– | 3·8 | 3·5 | 3·4 |

Males of the important age-group 50 to 69 make up a rather larger percentage of the population in England and Wales (9·3) than in Norway (8·3), while the position of Switzerland is intermediate (9·0) ; these small differences cannot account for the differences in the death-rates, and justify the use of crude death rates.]

TABLE IX.—*Cancer of Lung as Per cent of All Autopsies and of All Cancer Diagnosed at Autopsy in Different Countries and in Different Periods, Compared with Tobacco Consumption.*

| Locality. | Period. | Cancer of the lung as per cent of: All autopsies. Male. | Female. | Persons. | All cancer autopsies. Male. | Female. | Persons. | Annual tobacco consumption (lb. per head). |
|---|---|---|---|---|---|---|---|---|
| *Britain.* | | | | | | | | |
| 16 British hospitals* | 1894–1918 | .. | .. | 0·96–1·16 | .. | .. | .. | 1900–02, 1·9 (cgts. 0·6) |
| (Passey and Holmes) | 1919–1923 | .. | .. | 1·63 | .. | .. | .. | 1906–08, 2·0 ( „ 0·7) |
| | 1924–1928 | .. | .. | 2·11 | .. | .. | .. | 1911–13, 2·1 ( „ 0·9) |
| | | | | | | | | 1923–25, 2·9 ( „ 1·8) |
| Leeds General Infirmary* | 1891–1932 | .. | .. | 0·81–1·38 | .. | .. | 4·95–8·27 | 1929–31, 3·3 ( „ 2·4) |
| (Bonser) | 1933–1937 | .. | .. | 2·31 | .. | .. | 11·32 | 1934–36, 3·6 ( „ 2·7) |
| Western Infirmary, | 1925–29 | .. | .. | 2·9 | 16·5 | 14·5 | 15·7 | .. |
| Glasgow (Cappell) | 1930–34 | .. | .. | 3·0 | 20·2 | 6·1 | 15·3 | .. |
| | 1935–39 | .. | .. | 3·1 | 18·9 | 5·6 | 13·7 | 1936–37, 3·9 (cgts. 2·9) |
| | 1940–44 | .. | .. | 4·4 | 22·8 | 12·9 | 18·8 | .. |
| | 1945–48 | .. | .. | 5·4 | 24·1 | 14·3 | 20·4 | 1946–47, 5·0 ( „ 4·3) |
| St. Mary's, London | 1930–34 | 3·6 | 1·0 | 2·7 | 21·4 | 6·1 | 16·1 | .. |
| (Newcomb) | 1935–39 | 4·8 | 2·0 | 3·8 | 21·1 | 8·0 | 18·9 | .. |
| | 1940–44 | .. | .. | .. | .. | .. | .. | .. |
| | 1945–48 | 6·2 | 1·9 | 4·4 | 29·5 | 8·9 | 21·4 | .. |
| St. Bartholomew's, | 1930–34 | 5·4 | 2·3 | 4·3 | 27·7 | 13·7 | 23·3 | .. |
| London | 1935–39 | 3·7 | 1·9 | 3·1 | 19·3 | 8·2 | 15·0 | .. |
| (Cunningham) | 1940–44 | .. | .. | .. | .. | .. | .. | .. |
| | 1945–48 | 7·9 | 4·6 | 7·0 | 38·7 | 16·7 | 31·0 | .. |
| *Turkey.* | | | | | | | | |
| Istanbul (Saglam, | 1935–39 | 1·8 | 0·5 | 1·4 | 15·2 | 5·2 | 12·1 | 1937, 1·7 |
| Schwartz) | 1940–44 | 1·9 | 0·9 | 1·7 | 15·9 | 10·0 | 14·8 | 1942, 2·1 |
| | 1945–49 | 2·8 | 0·8 | 2·1 | 20·3 | 7·8 | 17·0 | 1947, 2·2 |
| *Yugoslavia.* | | | | | | | | |
| Ljubljana (Hribar, Kosir) | 1925–29 | 1·4 | 0·4 | 0·9 | 11·1 | 3·1 | 7·3 | .. |
| | 1930–34 | 0·6 | 0·1 | 0·4 | 4·3 | 1·1 | 2·9 | .. |
| | 1935–39 | 1·0 | 0·1 | 0·6 | 8·3 | 0·9 | 4·8 | .. |
| | 1940–44 | 1·7 | 0·1 | 0·9 | 14·0 | 0·7 | 7·2 | .. |
| | 1945–49 | 1·5 | 0·6 | 1·1 | 11·8 | 3·1 | 7·1 | .. |
| *Switzerland.* | | | | | | | | |
| Zürich (Meyenburg) | 1927–41 | .. | .. | 1·33 | .. | .. | 7·7 | 1936, 3·5 |
| *Iceland.* | | | | | | | | 1913 (cgts.) 0·03 |
| Reykjavik (Dungal) | 1932–48 | .. | .. | 0·6 | .. | .. | 2·9 | 1932 ( „ ) 0·3 |
| | | | | | | | | 1947 ( „ ) 1·4 |
| *Britain.* | | Most recent period. | | | | | | |
| Western Infirmary | 1947–48 | .. | .. | 5·8 | 30·1 | 13·2 | 23·5 | .. |
| St. Mary's Hospital | 1947–48 | 6·2 | 2·6 | 4·8 | 27·7 | 12·8 | 22·3 | 1947–48, 4·4 (cgts. 3·7) |
| St. Bartholomew's | 1947–48 | 8·3 | 3·2 | 6·8 | 41·7 | 14·8 | 33·3 | .. |
| *Turkey.* | | | | | | | | |
| Istanbul | 1949–50 | 4·1 | 0·8 | 2·8 | 29.8 | 7·5 | 22·3 | 1949, 1·9 |
| *Yugoslavia.* | | | | | | | | |
| Ljubljana | 1948–49 | 2·1 | 0·9 | 1·5 | 13·5 | 4·5 | 8·8 | .. |

* Figures refer to intrathoracic cancer.

TABLE X.—*Death Rates from Cancer of the Lung in England and Wales, Switzerland and Norway, Compared with Tobacco Consumption.*

| Year. | England and Wales.* Cancer of the lung. Death rates per million. M. | F. | Persons. | Annual tobacco consumption (lb./head). | Switzerland. Cancer of the lung. Death rates per million. M. | F. | Persons. | Annual tobacco consumption (lb./head). | Norway. Cancer of the lung. Death rates per million. M. | F. | Persons. | Annual tobacco consumption (lb,/head). |
|---|---|---|---|---|---|---|---|---|---|---|---|---|
| 1931 | 85 | 31 | 57 | 3·2 (cgts. 2·3) | 66 | 17 | 41 | ‡ | 14 | 9 | 12 | 1·4 (cgts. 0·5) |
| 1932 | 93 | 32 | 61 | 3·3 ( „ 2·4) | 62 | 19 | 40 | ‡ | 12 | 10 | 11 | 1·4 ( „ 0·5) |
| 1936 | 142 | 43 | 91 | 3·8 ( „ 2·8) | 86 | 21 | 52 | 3·5 | 18 | 20 | 19 | 1·7 ( „ 0·6) |
| 1937 | 161 | 43 | 100 | 4·0 ( „ 3·0) | 94 | 19 | 55 | 4·0 (cgts. 1939 1·4) | 28 | 20 | 24 | 1·9 ( „ 0·7) |
| 1940 | 222 | 55 | 131 | † | 115 | 31 | 71 | 4·6 | 31 | 18 | 24 | 2·2 ( „ 0·9) |
| 1943 | 321 | 60 | 173 | † | 162 | 33 | 95 | 4·9 | 39 | 24 | 31 | 1·0†( „ 0·5) |
| 1946 | 366 | 71 | 206 | 5·4 (cgts. 4·6) | 177 | 36 | 104 | 5·9 | 43 | 36 | 39 | 2·6 ( „ 1·1) |
| 1947 | 398 | 78 | 228 | 4·5 ( „ 3·8) | 183 | 34 | 106 | 5·7 (cgts. 1949 2·3) | 56 | 40 | 48 | 2·9 ( „ 1·3) |

* The figures available for tobacco refer to the United Kingdom, and those for cancer of the lung to England and Wales. If the smoking habits in Scotland and Northern Ireland are not very different from those in England and Wales, which contain 87 per cent of the population, this difference is of no importance.

† Disturbed by war.

‡ Not available.

Estimated population in thousands, Mid-year, 1947 (*Annual Abstract of Statistics,* No. 85 (1948).)

| | | |
|---|---|---|
| England and Wales | 43,050 | Great Britain, 48,189. |
| Scotland | 5,139 | |
| Northern Ireland | 1,350 | |
| United Kingdom | 49,539 | |

The data show that:

(1) The apparent incidence of cancer of the lung is increasing in both sexes in all three countries.

(2) The rate of increase in the 10 years 1936–7 to 1946–7 has been much the same in the three countries; in the five years 1931–2 to 1936–7 it was more rapid in Norway than in England and Wales, and was slowest in Switzerland.

*Death Rate from Cancer of the Lung.*

1936–7 as percentage of 1931–2.

| | England and Wales. | | | Switzerland. | | | Norway. | | |
|---|---|---|---|---|---|---|---|---|---|
| | M. | F. | Persons. | M. | F. | Persons. | M. | F. | Persons. |
| 1931–2 | 100 | 100 | 100 | 100 | 100 | 100 | 100 | 100 | 100 |
| 1936–7 | 170 | 137 | 162 | 141 | 111 | 132 | 177 | 211 | 187 |
| | | | | 1946–7 as percentage of 1936–7. | | | | | |
| 1936–7 | 100 | 100 | 100 | 100 | 100 | 100 | 100 | 100 | 100 |
| 1946–7 | 252 | 173 | 227 | 200 | 175 | 196 | 215 | 190 | 202 |

(3) The rate of increase has been greater for males than for females in England and Wales and in Switzerland.

(4) The incidence is comparatively low in Norway. In 1947 the crude death rate was approximately the same as it had been in England and Wales and in Switzerland 16 years previously.

| | Population (both sexes) producing one death from cancer of the lung. 1931. | 1947. |
|---|---|---|
| Norway | 83,300 | 20,800 |
| Switzerland | 24,400 | 9,400 |
| England and Wales | 17,500 | 4,400 |

(5) The difference is essentially a difference of incidence in men. The incidence in women is similar in Norway and Switzerland and about twice as great in England and Wales (Table X).

The incidence of cancer of the lung is much more nearly equal in the two sexes in Norway than it is in the other countries which have been studied.

| | Death rate from cancer of the lung in women as percentage of the death rate in men (1947). |
|---|---|
| Norway | 71% |
| England and Wales | 20% |
| Switzerland | 19% |

The ratios of cancer of the lung in women to total autopsies in women also indicates a relatively low incidence in women in Turkey and Yugoslavia (Table IX).

*The relation between increases in the consumption of tobacco, and in cancer of the lung.*

One should perhaps refer again, though there may be no need to do so, to the absence of any necessary connection between two quantities which are varying in the same way ; thus wireless licences in this country have multiplied at a rate very similar to that shown by cancer of the lung (Kennaway and Kennaway, 1947).

In Tables XI and XII comparisons are attempted of data from this country, Norway, and Switzerland during three periods, 1931–2, 1936–7 and 1946–7.

TABLE XI.—*Comparison between Tobacco Consumption, Cigarette Consumption and Death Rate (Persons) from Cancer of the Lung at Different Periods.*

| Country. | Tobacco consumption (lb. per head). | Cigarette consumption (lb. per head). | Death rate from cancer of the lung. |
|---|---|---|---|
| | 1946–7 as percentage of 1936–7. | | |
| England and Wales | 128 | 145 | 227 |
| Switzerland | 155 | 170* | 196 |
| Norway | 153 | 185 | 202 |
| | 1936–7 as percentage of 1931–2. | | |
| England and Wales | 120 | 123 | 162 |
| Norway | 129 | 130 | 187 |

* 1949 as percentage of 1939.

In all three countries the increase in cancer of the lung was greater than the increase in tobacco consumption between 1931–2 and 1936–7, and between 1936–7 and 1946–7. The increase in consumption of cigarettes was more like the increase in lung cancer over the later period, but there was a considerable gap between the figures in England and Wales.

TABLE XII.—*Comparison between Tobacco Consumption, Cigarette Consumption and Death Rate (Persons) from Cancer of the Lung in England and Wales and Norway, and in England and Wales and Switzerland.*

| Period. | Tobacco consumption (lb. per head). | Cigarette consumption (lb. per head). | Death rate from cancer of the lung. |
|---|---|---|---|
| | England and Wales as percentage of Norway. | | |
| 1946–7 | 180 | 350 | 499 |
| 1936–7 | 190 | 446 | 444 |
| 1931–2 | 232 | 470 | 513 |
| | England and Wales as percentage of Switzerland. | | |
| 1946–7 | 085 | 162 (1949) | 205 |
| 1936–7 | 104 | 266 (1939) | 179 |

The difference in death rates between England and Wales and Norway was also very much greater than the difference in tobacco consumption ; it was, however, reasonably close to the difference in cigarette consumption. In Switzerland tobacco consumption was much the same as in England and Wales, but death rates were only half as high. Differences in cigarette consumption again agree with the differences in death rates better than do differences in total tobacco consumption. This is because a much smaller proportion of the tobacco is consumed in the form of cigarettes in Switzerland than in England.

Clearly no exact proportionality has been found in the data so far available between the amount of tobacco smoked and the prevalence of cancer of the lung in different countries and at different periods. Cancer of the lung appears to increase more rapidly than does the use of tobacco ; such a change in effect may occur at a certain level in the dosage of a drug. A complicating factor is that when the population of a country consumes more tobacco, one does not know how much of this is due to the initiation of new smokers and how much to the increased use of tobacco by those who smoke already.

### DISCUSSION.

(1) The high proportion of cancer of the lung found among autopsies on cases of malignant disease in males in Istanbul suffices to show that the arsenic content of tobacco has not provided any simple and exclusive explanation of the association between cigarette smoking and this form of cancer. It is perhaps too soon to say that arsenic is quite unobjectionable in tobacco, for we have no information about the minimum effective dose of any carcinogen in man, nor about the possible summation of effect of different carcinogens.

As yet we have autopsy figures only from a single centre in Turkey, and of course more data from this and other sources, in Greece and Bulgaria, for instance, are very desirable. The figures from a single hospital in Yugoslavia suggest an incidence of cancer of the lung comparable to the British figures of 30 years ago. We have no figures for the incidence of cancer of the lung upon the whole population of any East European country. The official figures for the consumption of home-grown tobacco are liable to the error, that a portion may escape record.

(2) We do not know whether tobacco smoke itself contains some carcinogen, or whether the practice of smoking renders the respiratory tract more susceptible to agents from external (e.g. the benzpyrene of coal-smoke) or internal, sources. If the carcinogen is in the tobacco smoke, and is in particulate form, we facilitate its penetration to the bronchi by inhalation through the mouth, thus evading the nasal filter. The evidence available at the moment is against the direct carcinogenic importance of tobacco smoke, because it tends to exclude the two most obvious carcinogens, arsenic and benzpyrene, which one might expect to be present. But the range of chemical structure of known carcinogens is now so wide that one must consider other possibilities.

(3) All the older data about cancer of the lung must be reviewed in the light of this connection with smoking ; one must reconsider the inverse relationship with sunlight (Stocks, 1947) and the very low incidence upon a rather curious assortment of occupations, namely, agriculture, coal-mining, and mule-spinning (Kennaway and Kennaway, 1947). The coal-miner who works below-ground cannot smoke during one-third of the day, and the mule-spinner, and those in many other occupations, cannot smoke while at work.

(4) The incidence of cancer of the lung in Switzerland, and in Istanbul, where comparatively little coal-smoke would be expected, is against the importance of of this factor, but in this country (Stocks, 1936; Kennaway and Kennaway, 1947) and in Norway (see above) the mortality is higher in towns than in the country. In all such comparisons one must consider at least three possible factors, namely, (*a*) smoking habits, (*b*) facilities for diagnosis and treatment, and (*c*) atmospheric pollution with products of the combustion of coal tar, or of the internal combustion engine.

(5) The indication that cigarettes are more active than cigars and pipe tobacco in relation to cancer of the lung raises the question, whether this difference is due (*a*) to the method of combustion, or (*b*) to some property of cigarette tobacco. One cannot answer this question at present, but it is especially important in countries where smokers buy the cheaper pipe tobacco to make their own cigarettes. In Norway one obtains in this way nearly twice as many cigarettes for a given sum (Jakobsen, personal communication). One must consider three possibilities: (1) Pipe tobacco smoked in pipes; (2) pipe tobacco smoked in cigarettes; (3) cigarette tobacco smoked in cigarettes.

(6) The data given and discussed above from this country, Norway and Switzerland show that the study of the relations of national consumption of tobacco, and national incidence of cancer of the lung, has scarcely begun.

### SUMMARY.

(1) Estimations of arsenic in cigarettes from the United States, Canada, England, Norway, France, Switzerland, Italy, Austria, Poland and Bulgaria show on the whole a transition from the arsenic-rich American type in the West, to the arsenic-poor Turkish type in the East; the latter is, of course, smoked in Western countries also.

(2) In Yugoslavia, Turkey, Greece and Bulgaria, the tobacco consumed is almost wholly home-grown, of Turkish type, and in the form of cigarettes.

(3) The high incidence of cancer of the lung at autopsy in one centre (Istanbul), in a country where Turkish tobacco is smoked almost exclusively, shows that the arsenic content of tobacco has not provided any simple and exclusive explanation of the association between cigarette smoking and this form of cancer. Cancer of the lung appears to be much less frequent at a centre in Yugoslavia (Ljubljana) than at Istanbul. More information from these and other Balkan countries is very desirable.

(4) Norway imports about 85 per cent of the tobacco consumed from the U.S.A., and 9 per cent from the Balkan countries. The incidence of cancer of the lung upon the two sexes is not very different (death rate, male to female, 1 : 0·7); it is greater in urban than in rural districts, and this difference is greater in men than in women. The increase in mortality in the last 20 years has been greater in men than in women, and greater in the towns than in the country.

(5) In Sweden, snuff makes up a much larger fraction (one-third in 1949) of the total tobacco products consumed than is recorded in other countries.

(6) A comparison is made of the data available for the increases since 1931 in (*a*) deaths attributed to cancer of the lung, and (*b*) in the consumption of tobacco, in England and Wales, Norway and Switzerland. The consumption of tobacco per head has been for the last 10 years rather higher in Switzerland than in the United Kingdom, and in Norway has been about one-half that in the other two countries, while the crude death rates at the beginning and end of the period were roughly in the proportion of 10 (England and Wales) to 5 (Switzerland), and 2 (Norway). Cigarette consumption was approximately in the proportion of 4 (England and Wales) to 2 (Switzerland) and 1 (Norway) and was more in accord with the relative death rates. The increase in the number of deaths has been about the same (twofold) in all three countries, but the increase in consumption of tobacco and cigarettes has been less. The differences in the incidence of cancer of the lung are therefore quite different in extent from those in the quantity of tobacco consumed ; they are more like (though still different from) those in the quantity of cigarettes consumed. The study of the relation between the national consumption of tobacco and the national incidence of cancer of the lung has scarcely begun.

We wish to thank Prof. Saglam and Prof. P. Schwartz, of the University of Istanbul, and Prof. Hribar, Director of the Institute of Pathological Anatomy, and Dr. A. Kosir, of the Clinical Hospital, Ljubljana, for their generosity in allowing us to use their very valuable records. We are indebted to Prof. Leiv Kreyberg, of Oslo, Mr. Olaf Jakobsen, of the Norwegian Embassy, Prof. H. R. Schinz, of the Kantonspital, Zürich, Prof. H. v. Meyenburg, of the Pathological Institute, Zürich, Mr. S. T. Hisim, of the Turkish Embassy, Mr. Popov, of the Bulgarian Legation, Prof. Elis Berven, of Stockholm, Prof. H. E. Rawlinson, of the University of Alberta, the Bureau Fédéral de Statistique, Berne, and the Direction Général des Douanes, Berne, for their kindness in supplying us with statistical and other material. In this country we have to thank Dr. G. J. Cunningham, Prof. D. F. Cappell, Prof. A. Bradford Hill, Prof. W. Newcomb, Dr. C. C. Spicer, Dr. Percy Stocks, Mr. H. L. Henderson, Mr. R. E. Waller and Miss Mary Atkin, for help in various ways. We wish to express our gratitude to the British Empire Cancer Campaign and the Anna Fuller Fund for generous grants.

### REFERENCES.

Bradford-Hill, A., and Faning, E. L.—(1948) *Brit. J. indust. Med.*, **5**, 2.
Chief Inspector of Factories and Workshops.—(1939) *Ann. Rep.*, p. 22. London (H.M. Stationery Office).—(1943) *Ibid.*, p. 45.
Currie, A. N.—(1947) *Brit. med. Bull.*, **4**, 402.
Daff, M. E., and Kennaway, E. L.—(1950) *Brit. J. Cancer*, **4**, 173.
Doll, R., and Bradford-Hill, A.—(1950) *Brit. med. J.*, ii, 739
Dungal, N.—(1950) *Lancet*, ii, 245.
Heady, J. A., and Kennaway, E. L.—(1949) *Brit. J. Cancer*, **3**, 311.
Henry, S. A.—(1950) *Ann. Roy. Coll. Surg. England*, **7**, 425.
Hutson, J. B.—(1937) *U.S. Dept. of Agric. Technical Bulletin No.* 587.
Kennaway, E. L., and Kennaway, N. M.—(1947) *Brit. J. Cancer*, **1**, 260.
Montgomery, H., and Waisman, M.—(1941) *J. Invest. Derm.*, **4**, 365.
Neubauer, O.—(1947) *Brit. J. Cancer*, **1**, 192.
Saglam, T.—(1944) *Bull. Fac. Med. Istanbul.* Yil : 7, Sayi : 2, Imumi No. 28, Nisan-Haziran, 1944, p. 3793.
Semon, H. C.—(1945) *Proc. Roy. Soc. Med.*, **38**, Section of Dermatology, 128.
Stocks, P.—(1936) *Ann. Rep. Brit. Emp. Cancer Camp.*, **13**, 239.—(1947) ' Studies on Medical and Population Subjects No. 1. Regional and Local Differences in Cancer Death Rates.' London (H.M. Stationery Office).
Wynder, E. L., and Graham, E. A.—(1950) *J. Amer. med. Ass.*, **143**, 329.